# Supplementary material for: Reputation shortcoming in academic publishing
Source: PLoS One. 2025 Apr 29;20(4):e0322012. doi: 10.1371/journal.pone.0322012 (PMC12040217; doi:10.1371/journal.pone.0322012)
Supplement: S1 File — References with § in the main text refer to the corresponding paragraph of this file. (DOCX) [file pone.0322012.s001.docx]

**Supplementary materials for “Reputation shortcoming in academic publishing”**

*Rémi Neveu, PhD; André Neveu, PhD*

**Table of contents**

[1. Supplementary methods 9](#_Toc184755103)

[1.1. Additional general considerations 9](#_Toc184755104)

[1.1.1. Overview 9](#_Toc184755105)

[1.1.2. Inclusion criteria of the prospective study 9](#_Toc184755106)

[1.2. Methodological considerations 9](#_Toc184755107)

[1.3. Inclusion criteria 10](#_Toc184755108)

[1.3.1. Additional considerations for journals and editors 10](#_Toc184755109)

[1.3.2. Job offers 11](#_Toc184755110)

[1.4. Data collected 11](#_Toc184755111)

[1.4.1. Nature journals 11](#_Toc184755112)

[1.4.2. Editors’ data 11](#_Toc184755113)

[1.4.2.1. Computation of the arrival date of the editor at the current Nature journal 14](#_Toc184755114)

[1.4.2.2. Decisional algorithm to assess the presence of any research experience after the doctorate in each editor’s biography: 15](#_Toc184755115)

[1.4.3. Journal impact factor 16](#_Toc184755116)

[1.4.4. Retracted articles 17](#_Toc184755117)

[1.4.5. Job offers 17](#_Toc184755118)

[1.4.6. Submissions 18](#_Toc184755119)

[1.5. Definitions 19](#_Toc184755120)

[1.5.1. New authors 19](#_Toc184755121)

[1.5.2. Original articles, reviews and meta-analyses 20](#_Toc184755122)

[1.6. Data preprocessing and computation 21](#_Toc184755123)

[1.6.1. Shared step for the extraction of article data for Nature journals and editors 21](#_Toc184755124)

[1.6.2. Extraction of article data for Nature journals 23](#_Toc184755125)

[1.6.3. Extraction of article data for editors 23](#_Toc184755126)

[1.6.4. Extraction of data for retracted articles 25](#_Toc184755127)

[1.6.5. Handling of missing or incomplete data 25](#_Toc184755128)

[1.6.6. Handling of homonyms 27](#_Toc184755129)

[1.6.6.1. Building groups of articles 27](#_Toc184755130)

[1.6.6.2. Identifying homonyms among editors and authors 27](#_Toc184755131)

[1.6.6.3. Dates 31](#_Toc184755132)

[1.6.7. Homonyms among authors of articles published in Nature journals 31](#_Toc184755133)

[1.6.8. Year of launch of Nature and Science journals 32](#_Toc184755134)

[1.6.9. Handling of programming errors 32](#_Toc184755135)

[1.6.10. Duration of the periods before and after the editors’ appointment used to investigate conflicts of interests 32](#_Toc184755136)

[1.7. Data analyzed 34](#_Toc184755137)

[1.7.1. Editors 34](#_Toc184755138)

[1.7.2. Journals 35](#_Toc184755139)

[1.7.3. Articles 35](#_Toc184755140)

[1.7.4. Job offers 36](#_Toc184755141)

[1.8. Material 36](#_Toc184755142)

[1.9. Statistical analyses 36](#_Toc184755143)

[1.9.1. Computations 36](#_Toc184755144)

[1.9.1.1. Quantification of the scientific and editorial experiences of editors 36](#_Toc184755145)

[1.9.1.2. Nature journals 37](#_Toc184755146)

[1.9.2. General statistical considerations 38](#_Toc184755147)

[1.9.3. Statistical models and tests 38](#_Toc184755148)

[1.9.3.1. Analysis of the number of articles published by editors’ former co-authors and the other authors in the editor’s journal 39](#_Toc184755149)

[1.9.3.2. Quantification of chances to publish in the editor’s journal as a function of the author’s publishing record (or experience in publishing) in this journal and the author’s status to the editor (i.e. former co-authors vs other authors) 42](#_Toc184755150)

[1.9.3.3. Analysis of the total number of articles published by authors with the same affiliations as the editor when he/she was working as a researcher 43](#_Toc184755151)

[1.9.3.4. Analysis of the dynamics of the articles published by new authors and known intermediate authors 45](#_Toc184755152)

[1.9.3.5. Association between the journal’s impact factor and differences in publications between new authors and established authors 47](#_Toc184755153)

[1.9.3.6. Comparison of changes in the number of publications by editors’ co-authors and the other authors before and after the setup of the non-financial conflicts of interest policy at Nature journals 50](#_Toc184755154)

[1.9.3.7. Comparison of changes in the number of publications by authors with the same research affiliation as the editor’s previous ones in the editor’s journal and the other Nature journals before and after the setup of the non-financial conflicts of interest 52](#_Toc184755155)

[1.9.4. Limits of our analyses 53](#_Toc184755156)

[1.10. Additional statistical analyses and models 58](#_Toc184755157)

[1.10.1. Characteristics of editors of Nature journals 58](#_Toc184755158)

[1.10.2. Analysis of the number of articles published by new authors at the launch of a new Nature journal 58](#_Toc184755159)

[1.10.3. Association between the journal impact factor at year n-2 and the rate of articles published by new first and last authors in generalist and specialized Nature journals 59](#_Toc184755160)

[1.10.4. Analysis of retracted articles 60](#_Toc184755161)

[1.10.5. Analysis of job offers for editorial positions at Nature journals 62](#_Toc184755162)

[1.10.6. Association between journals impact factor and the prestige of Nature journals 63](#_Toc184755163)

[1.10.7. Analysis of the consequence of a change in submission behavior of authors over their publications in the editor’s journal 64](#_Toc184755164)

[1.10.8. Comparison of the publication record in the editor’s journal of editors’ co-authors and the other authors before the editor’s appointment 66](#_Toc184755165)

[1.10.9. Analysis of the association between the recruitment date of the editor and the increase in publications of editor’s co-authors and the other authors in the editor’s journal 66](#_Toc184755166)

[1.10.10. Analysis of the association between the recruitment date of the editor and the number of articles published by editor’s co-authors and the other authors before the editor’s appointment 68](#_Toc184755167)

[1.11. Control and robustness analyses 68](#_Toc184755168)

[1.11.1. Control analyses 68](#_Toc184755169)

[1.11.2. Robustness analyses 69](#_Toc184755170)

[1.12. Specific considerations for the identification of editors without any research experience after the doctorate, the identification of conflicts of interests and the analysis of retracted articles 70](#_Toc184755171)

[2. Supplementary results 71](#_Toc184755172)

[2.1. Additional results of analyses reported in the main text 71](#_Toc184755173)

[2.1.1. First goal of the analysis reported in §1.9.3.1 71](#_Toc184755174)

[2.1.2. Second goal of the analysis reported in §1.9.3.1 71](#_Toc184755175)

[2.1.3. Analysis reported in §1.9.3.3 72](#_Toc184755176)

[2.1.4. Analysis reported in §1.9.3.4 72](#_Toc184755177)

[2.1.5. Analysis reported in §1.9.3.5 73](#_Toc184755178)

[2.1.6. Analysis reported in §1.9.3.7 74](#_Toc184755179)

[2.2. Results of additional analyses 74](#_Toc184755180)

[2.2.1. Nature journals have only professional editors. 74](#_Toc184755181)

[2.2.2. IF is associated with the prestige of the journal 74](#_Toc184755182)

[2.2.3. The launch of a new Nature journal is not associated with more articles published by only new authors 75](#_Toc184755183)

[2.2.4. The current recruitment policy of editors at Nature journals contributes to increase rather than decrease the differences in publications between authors with different reputation to the editors 76](#_Toc184755184)

[2.2.4.1. Requested characteristics for candidates applying for job offers for editorial positions at Nature journals and association with the characteristics of the editorial board 77](#_Toc184755185)

[2.2.4.2. Relationship between the launch of new journals and the recruitment of editors 77](#_Toc184755186)

[2.2.4.3. Editors who stay at Nature journals have a lower scientific experience than the newly appointed ones 78](#_Toc184755187)

[2.2.4.4. Comparison of the publication record of editor’s former co-authors before the editor’s appointment with the record of the other authors 78](#_Toc184755188)

[2.2.5. The attention paid to the content of the articles published in Nature journals may not contribute to the differences in publications observed between authors with distinct reputational inputs to the editors 78](#_Toc184755189)

[2.2.6. Editors of Nature journals are requested to liaise extensively with researchers 79](#_Toc184755190)

[2.3. Results of the robustness analyses 80](#_Toc184755191)

[2.4. Results of analyses mentioned only in the supplementary materials 80](#_Toc184755192)

[2.4.1. Prestige of Nature journals in job offers for editorial positions at Nature journals 80](#_Toc184755193)

[2.4.2. Relationship between the rate of original articles of new first and last authors and subsequent IF 81](#_Toc184755194)

[2.4.3. Justification of the consideration of all articles published in all Nature journals in the definition of new and known authors and justification of not tying editors to the manuscripts they handled 81](#_Toc184755195)

[2.4.4. Justification of the consideration of the training period in the exclusion period of publication after the editor’s appointment 82](#_Toc184755196)

[2.4.5. Lack of association between a change in submission behavior of authors and the increase in publications of editor’s former co-authors 82](#_Toc184755197)

[2.4.6. Lack of association between the increase in publications of editor’s co-authors (respectively of the other authors) after the editor’s appointment and the time elapsed since the editor’s appointment 83](#_Toc184755198)

[2.4.7. Lack of association between the number of articles published by editor’s co-authors before the editor’s appointment and the time elapsed since the editor’s appointment 84](#_Toc184755199)

[3. Supplementary figures 86](#_Toc184755200)

[3.1. Supplementary figure 1 86](#_Toc184755201)

[3.2. Supplementary figure 2 87](#_Toc184755202)

[3.4. Supplementary figure 3 89](#_Toc184755203)

[4. Supplementary references 91](#_Toc184755204)

# Supplementary methods

## Additional general considerations

### Overview

We complemented the main longitudinal retrospective study by a prospective one and a second retrospective longitudinal study. The prospective study focused on job offers for editorial positions at *Nature* journals. It included *Nature* journals’ job offers for editorial positions issued on the *Nature* website between December 2020 and December 2021. The second longitudinal retrospective study focused on retracted articles in the journals included in the main study (supp. §1.4.4). These articles were extracted from the PubMed database directly through the PubMed website while the main study used compressed files containing the entire database. The rationale of using the website rather than building programs to extract articles from the database is the limited amount of data that had to be extracted: PubMed allows to extract .csv files that do not contain more than 10000 references.

These additional studies complied with the French law protecting participants to biomedical research and with the European Union rules protecting personal data.

### Inclusion criteria of the prospective study

Job offers were included in the study if they referred to editorial positions:

1. at *Nature* journals included in the study
2. aiming at making editorial decisions about submitted manuscripts or comments (see §1.3).

The inclusion flow chart is depicted in supplementary figure 1.

## Methodological considerations

To control for methodological biases, we did not recruit editors directly (i.e. through volunteering participation). Editors would have had a conflict of interest in accepting to self-report biases in their editorial decisions. Conflicts of interests are better detected among reviewers with the method we used than with self-reports *[1]*. Editors who publish articles as co-authors disclose less their conflicts of interests than other authors *[2]*.

Our study was compatible with the French law protecting participants of biomedical research *[3].* It did not have to be evaluated by the adequate institutions (Comité pour la Protection des Personnes). It also respected the considerations of the Helsinki declaration *[4]* which were applicable to our study design. We checked that the terms and conditions of the *Nature* [5], *LinkedIn* [6] and *ORCID* [7] websites allowed us to collect the data for the goal of this study. Additionally, because we used publicly accessible data in the context of a scientific study reporting aggregated data, because of the type of data we collected and because of the methodological considerations to control for biases, our study complies with the European Union’s General Data Protection Rules [8] and their application in France [9-11].

## Inclusion criteria

### Additional considerations for journals and editors

The rationale of targeting original articles rather than all types of articles, such as reviews published in journals whose name start with “*Nature Review*”, is that:

- these articles report new results and discoveries as compared to other articles such as reviews, meta analyses, commentaries, opinion articles…

- they shape substantially the current research efforts across the world,

- they require larger funding than the other types of articles especially because of the experimental parts.

The use of the stable of *Nature* journals has other several methodological advantages for a systematic and in-depth analysis of editorial decisions: journals are either generalist or specialized and span almost all research areas; all editors are professionals [12], have connections with the other *Nature* journals’ editors (supp. §2.4.3 and [12]) and work full-time for the journal they are attached to [12, 13], removing thus confounds related to a simultaneous activity of academic research [14] highlighted by *Nature* itself [12]. As a comparison, the family of *Science* journals has a more heterogeneous editorial setup, composed mainly of a board of academic researchers with a minority of professional editors. It is the same for the family of *Cell* journals. Examining the *Nature* family of journals provides a larger dataset than other family journals (i.e. *Science*, *Cell*, *PNAS*…). The family of *Science* journals comprises only 6 journals and 45 professional editors. Therefore, including other journal families like *Science* would be a major confounding factor. Additionally, the dataset would be unbalanced between journal families. This would weaken the statistical results.

### Job offers

Job offers were included in the study if they:

1. were released by the *Springer Nature* group
2. were labeled in the *Nature* website as “publishing or editorial” positions
3. were registered in the *Nature Research* section in the *Nature* website.

## Data collected

### Nature journals

*Nature Italy*, *Nature Digest* and *Nature Protocols* were excluded because *Nature Italy* focuses on Italian research, *Nature Digest* is in Japanese and *Nature Protocols* publishes articles of secondary research.

For the 33 *Nature* journals, we collected their year of launch on the *Nature* website (https://www.nature.com/siteindex) or Wikipedia (https://fr.wikipedia.org).

### Editors’ data

From November 14 2020 to December 15 2020, we manually retrieved, for each *Nature* journal included in the study (table 1), information of the editors’ biography from the dedicated webpage of the *Nature* journal. This webpage is entitled “About the editors”. It provides a short biography of the editors of the journal. It has an URL construct https://www.nature.com/XXXX/about/editors with XXXX representing an acronym of the name of the journal. We collected:

1. The full name of the editor
2. The name, city and country of the research institutions where the editor worked as a researcher (during the doctorate (PhD or MD) or after) before being appointed at *Nature* journals
3. The name of the principal investigator of the laboratory where the editor worked if available
4. The arrival date of the editor at their current journal and its name
5. Whether the editor had an editorial experience in another *Nature* journal before. In that case, the date when the editor was appointed at one of the *Nature* journals (i.e. journals whose name starts with “*Nature*” even if these journals were not included in the study) for the first time was retrieved from the *Nature* website, the editor’s LinkedIn profile, the editor’s ORCID profile or a direct search on Google. This date was computed following the same method as the arrival date of the editor in the current *Nature* journal
6. Whether the editor had only a PhD or MD experience or whether it was completed by any research experience after the doctorate
7. The type of editorial position: editors making decisions about submitted primary research articles (i.e. such decisions are referred as “selection and handling of the manuscripts” in *Nature* journals’ job offers), editors making decisions about comments made by other researchers on articles published in the *Nature* journals and editors managing other types of articles (e.g. news and views section, multimedia section, career section…).

We complemented these data with data collected from LinkedIn, ORCID and Google from January 23 2021 to January 25 2021. Direct searches on Google typically retrieved universities websites where the editor worked previously as a researcher or websites where the editor gave a talk. LinkedIn is a website dedicated to disclose CVs where each member is responsible for the disclosed content of his/her CV and must not disclose wrong or biased information [6]. Anybody can freely become a member of LinkedIn and disclose his/her CV [6]. ORCID is a website dedicated to identifying each researcher uniquely and is based on volunteering like LinkedIn. The ORCID’s main goal is to identify publication associated with a specific person and prevent biases because of homonyms. ORCID also allows to disclose his/her professional experience. We collected for each editor:

1. The starting year of the doctorate (PhD or MD), the ending year of the doctorate (PhD or MD), the last year during which the editor worked as a researcher in a research institution.
2. The number of different research laboratories where the editor worked before joining *Nature* journals
3. The arrival date at *Nature* journals and the name of the first *Nature* journal where the editor worked whenever the editor had a previous editorial experience in another *Nature* journal. This date was computed with the same method as for the arrival date in the current *Nature* journal where the editor works.

LinkedIn, ORCID and Google were also used to collect data which were missing from the *Nature* website and to check that data were consistent. The match between the *Nature* website and the other websites (LinkedIn, Orcid or results from a Google search) was made with:

1. the name and first name of the editor
2. the statement of being editor at *Nature* journals in the *Nature* journal where the editor works at the date of the data collection,
3. and the inclusion of the date range of the data collection in the dates of the editor’s CV reported in the other websites if the information was available.

The statement of working as editor at *Nature* journals was set to prevent homonyms in the matching of the *Nature* website with the other websites. Whenever this match could not be done with the information of being editor at *Nature* journals, we used the academic research labs where the editor worked before being recruited by *Springer Nature* whenever these labs were mentioned on the *Nature* website. If the labs were not mentioned, the match could not be performed and only the data collected from the *Nature* website were included in the study.

One editor had information disclosed on the *Nature* website which were inconsistent with the ones of LinkedIn. These information were the academic research labs where the editor worked before being recruited by *Springer Nature*. However, the information related to the editorial positions at *Nature* journals were consistent. Because LinkedIn webpages are written by the editor themselves while the editor’s biography on the *Nature* website may not have been written by the editor himself, we replaced the information collected on the *Nature* website by the ones of LinkedIn for that editor.

All webpages of the *Nature* journals, LinkedIn (https://www.linkedin.com), ORCID (https://orcid.org/) and webpages referenced in Google used to retrieve data are open access.

#### Computation of the arrival date of the editor at the current Nature journal

The precision of the dates retrieved was very heterogeneous. Some had the year and the month. Others had the year and the season (winter, spring, summer and autumn). Some had the year and the semester. Others had only the year. This heterogeneity may have biased the results if data had been analyzed without any caution: for instance, if we retrieved the year and the semester for the arrival date of an editor, one could not know when that editor started to process articles submitted to the journal during that semester. We therefore excluded any article which had been published during that period or during the same period shifted by several months depending on the statistical analysis. Basically, this shift aimed at taking into account the duration of the publication process from the initial submission to the publication in the journal (see the third step in §1.6.3). This exclusion period was therefore equal to: one month if the year and the month of the arrival date of the editor were retrieved, three months if the year and the season were available, six months if the year and the semester were available, and one year when only the year could have been retrieved. This exclusion period was used in all analyses involving directly editors (analyses assessing conflicts of interests related to editors’ former co-authors and research institutions where editors worked before being appointed at *Nature* journals). In practice, for an editor who joined *Nature* journals in February of a given year, the exclusion period was the entire month of February of that year; for an editor who joined *Nature* journals during the summer, the exclusion period was from July to September of that year; for an editor who joined *Nature* journals during the second half of a year, the exclusion period ran from July to December of that year; and for an editor who joined *Nature* journals at a given year, the exclusion period was from January to December of that year.

In the case of a shift of the exclusion period, the lower-bound month and the upper bound month of that period were shifted by the corresponding number of months of the shift:

Shift

Lower bound

Upper bound shifted

Lower bound shifted

Upper bound

Time

Exclusion period shifted

Exclusion period during which the editor arrived at Nature (e.g. February, Summer…)

Thus, in the case of a shift of four months of a restriction time period ranging from January to March, articles published between May and July of the same year were discarded from the analyses. This sift was performed in all analyses requiring to take into account the duration of the publication process.

In analyses of articles published during a specific time window, e.g. the two years after the editor’s arrival in the journal, this time window ran, in that example, from the end of the aforementioned exclusion period. Similarly, if the time window was before the editor’s arrival at *Nature* journals, this time window ended at the beginning of the exclusion period.

#### Decisional algorithm to assess the presence of any research experience after the doctorate in each editor’s biography:

When the word “PhD” or “MD” was mentioned in the editor’s biography, we checked that this referred to the editor. To check for a research experience, we looked whether the word “postdoc”, “research assistant”, “group leader”, “researcher”, “professor” or any other description of a position referring explicitly to a research topic on which the editor worked in that position, was mentioned in the editor’s biography. If so, a statement had to explicitly show that the experience occurred after the doctorate (PhD or MD). Whenever there were research experiences that could not be explicitly associated with a research position after the doctorate, we checked in the editor’s LinkedIn profile (https://www.linkedin.com). An editor who did not mention any experience between the end of his doctorate position and the start of his position at *Nature* journals was considered an editor without any research experience after the doctorate (PhD or MD). If the editor’s LinkedIn profile was not available, we did the same with ORCID (https://orcid.org/). If the ORCID profile was not available, we looked on Google with the full name of the editor followed by the words “editor” and “*Nature*” whether there was any biography of the editor available on the website of a research institution. The rationale of this last search is that editors at *Nature* journals are requested to liaise extensively with researchers, laboratories or research institutions through conferences and visits as mentioned in 91% of the *Nature* journals’ job offers for any editorial position requiring to make decisions about submitted manuscripts in *Nature* journals (editor in chief, senior, associate or assistant editor, team manager (for *Nature Communications*)) and taken into account in the analyses (supp. §2.2.6). Sources of information were screened in the following order: *Nature* website first, LinkedIn second, ORCID third and Google fourth. In the case of discrepancies between two sources of information, a third source (among the previous list) was screened if possible. If a third source could not solve the discrepancy, the information with the highest level of details was kept. If there were still unsolved discrepancies between the four sources of data, the best information in favor of the *Nature* journal was kept.

If it appeared on the *Nature* website that the editor did not have any research position after the doctorate (PhD or MD), a systematic screening of LinkedIn, ORCID and Google was conducted to find whether any research experience after the editor’s doctorate (PhD or MD) was not mentioned. This systematic screening aimed at avoiding a wrong categorization (i.e. with/without a research experience after the doctorate) of editors.

### Journal impact factor

The journal impact factor (IF) history was downloaded for each *Nature* journal from Clarivate (formerly the ISI web of Science, https://clarivate.com/webofsciencegroup/solutions/journal-citation-reports/?subsector=18127). The history of the IF was available from 1997 (for the oldest *Nature* journals) until 2019. The IF of a given year is released around June of the following year. Thus the 2020 IF was not available at the time of the analyses. The IF is the average number of citations, at a given year, of the articles of a journal which have been published during the two years before [15].

### Retracted articles

On June 16 2021, for each *Nature* journal, we extracted from the PubMed website the retracted articles. In the advanced search of PubMed, we specified the journal name in the journal field and the date range January 1^st^ 1990-present in the publication date field. We filtered the results in order to keep only “retracted publications” and “retractions of publications”. These latter correspond to retraction notes which are published at the time of the retraction. Note that not all retracted articles have an associated retraction notes. The PubMed website generated a .csv file for each journal with the retracted articles and retraction notes published in the journal. Because the .csv file contained only the initials of the first name of authors, we manually checked with the PubMed website any set of two retracted articles (included in the analyses) where all authors’ first name initials and last name matched. This check aimed at removing duplicates in retracted articles and retraction notes.

### Job offers

From December 2020 to December 2021, we systematically collected every 17 days on average (SD=6.5 days) all job offers released by the *Springer Nature* group for the brand “*Nature* *Research*” and for “publishing/editorial” jobs. *Nature* journals’ job offers were available at:

<https://group.springernature.com/gp/group/careers/editorial/our-jobs>

and, starting from July 24^th^ 2021, at:

<https://career5.successfactors.eu/career?company=C0001215517P&career_ns=job_listing_summary&navBarLevel=JOB_SEARCH>. The *Nature* *Research* brand includes job offers for editorial positions at *Nature* journals but at other journals whose name does not start by *Nature* (e.g. “*Communications biology*”). In each job offer, we extracted:

1. The reference number of the job offer to allow a unique identification in case this offer was advertised a second time,
2. The publication date of the job offer,
3. The closing date for applications,
4. The date of collection of the job offer,
5. The type of position the job offer is for (associate/senior editor, editor in chief, team manager),
6. Whether the position was full time or required more than 35 hours of work per week. Whenever this was not mentioned, we screened for an explicit statement that the position was part-time or required less than 35 hours of work per week
7. Whether the position involved the “handling of submitted manuscripts” (which involved making decisions about the submitted manuscripts),
8. Whether applicants had to have a doctorate (PhD or MD),
9. Whether a postdoc experience was preferred or was not essential,
10. Whether a strong (or higher or significant) research record was preferred or was not essential,
11. Whether research experience should be up to a doctorate (PhD or MD) level or equivalent,
12. Whether the position would involve interactions with editors of other *Nature* journals,
13. Whether the position would require to meet or liaise with researchers, visit laboratories or institutions to learn about their research and make *Nature* journals better known,
14. Whether an editorial training would be provided,
15. Whether the job offer said that the journal publishes the world’s best research articles; the journal is the world’s leading multidisciplinary journal; the journal is the world’s leader in publishing high-quality scientific articles, the most significant or exceptional scientific advances, or articles of the highest quality and significance; the journal has a high impact and publishes high-quality articles,
16. The number of times the same job offer was published. A job offer was considered published again if the publication date of the new release of the job offer (i.e. same reference number as the previous release) was at least one day after the closing date for application of candidates for the previous release of the job offer.

### Submissions

Aggregated data of submitted original articles were publicly available on the *Nature* website (https://www.nature.com/nature/for-authors/editorial-criteria-and-processes). We retrieved them on November 6th 2022. They cover the period from 1997 to 2017. These data were the number of articles submitted to *Nature* as original articles each year. They also included the number of original articles published each year by *Nature*. We could not find similar data publicly available for the other *Nature* journals.

## Definitions

### New authors

This definition applied to all analyses in which we investigated new authors. Thus the definition did not apply to editors’ co-authors in analyses assessing conflicts of interests between editors and editors’ former co-authors.

An author of a given article was considered new regarding all *Nature* journals included in the study (table 1) if this author had not published any article of any kind in any *Nature* journal included in the study over N years before the publication of the given article. In the results reported in the main text, N was set to two years to have a consistent duration with the duration used to compute the impact factor of the journal. To assess the robustness of our results, we also ran all analyses for N=1, 3, 4, 5, 10 and +∞ years (i.e. since the launch of the journal). The rationale of including all *Nature* journals in the definition is that *Nature* journals’ editors are requested to liaise extensively with researchers, laboratories or research institutions through conferences and visits as mentioned in 91% of the *Nature* journals’ job offers for any editorial position requiring to make decisions about submitted manuscripts (editor in chief, senior, associate or assistant editor, team manager (for *Nature Communications*), see §2.2.6).

Interaction between authorship and this definition:

The authorship position (first or last vs intermediate) in an article discriminates the contribution of the author to the article (supp. §2.6.1). As a consequence, if all authors of a given article are ‘new’, then we used the phrase “article of *only new authors*”. *Known intermediate (or middle) authors* are set of authors of a given article that were neither first nor last authors (i.e. “intermediate” or “middle”) and who had published at least one article in any of the *Nature* journals included in the study for the two years preceding publication (i.e. “known”). This set of authors may not include all intermediate authors. Some intermediate authors may be ‘new’.

### Original articles, reviews and meta-analyses

Only original articles of the PubMed database were considered for the analyses of articles published by *Nature* journals. However, for the computation of the editors’ scientific experience, we considered all types of articles published in any journal referenced in the PubMed database.

Article labels in PubMed are available at https://www.nlm.nih.gov/mesh/pubtypes.html. Articles were considered original articles if:

- they had an abstract,
- they had one of the PubMed labels listed below,
- and if they had at least two authors.

The rationale for adding the constraint of two authors was that the label “Original article” does not exist in the PubMed database: the closest label in PubMed, i.e. “Journal article”, may include articles which do not include new datasets or new techniques. Articles with only one author which would include a new dataset or a new technique were expected to be extremely scarce due to the huge amount of work requested: thus most of the articles with only one author and the label “Journal article” would likely not have fitted the expectations of an original article.

The PubMed labels (available at https://www.nlm.nih.gov/mesh/pubtypes.html) used to characterize an original article were:

- Academic dissertation,
- Adaptive clinical trial,
- Clinical trial,
- Comparative study,
- Controlled clinical trial,
- Equivalence trial,
- Evaluation study,
- Journal article (only articles with at least two authors),
- Multicenter study,
- Observational study,
- Pragmatic clinical trial,
- Randomized controlled trial,
- Statistics,
- Technical report,
- Twin study,
- Validation study.

These labels were selected for their potential to refer to an original article. Note that an article referenced in PubMed may have more than one of these labels.

For reviews and meta-analyses, we used the same definition as for original articles except that the labels were:

- Meta-analysis,
- Review,
- Systematic review.

Because PubMed does not reference systematically all old articles and the labeling of original articles published in *Nature* was more hazardous before January 1^st^ 1990 (results and analyses are not shown here but are available at <https://entrepot.recherche.data.gouv.fr>: <https://doi.org/10.57745/3QL466>), all analyses focusing on articles published in *Nature* journals included articles published after January 1^st^ 1990. This restriction impacted only articles published in *Nature* but not the other *Nature* journals since they were launched after January 1^st^ 1990.

## Data preprocessing and computation

### Shared step for the extraction of article data for Nature journals and editors

The PubMed database was built of 1062 compressed files, each file containing 30000 references (20051 for the last file). We first extracted:

- all articles published in the *Nature* journals included in the study (table 1) sorted by *Nature* journal,
- and all articles, without any restriction over the journal, published by the editors of the *Nature* journals included in the study.

For each of these articles, we collected:

- the date of publication, i.e. year, month and day if available (from the field <PubDate>),
- the name of the journal (from the field <Title> inside the field <Journal>),
- first name (from the field <ForeName>), name (from the field <LastName>) and initials (from the field <Initials>) if available for all authors of the article,
- the affiliation of each author of the article (from the field <Affiliation> inside the field <AffiliationInfo>),
- the ORCID identifier of each author of the article (from the field <Identifier>),
- whether the list of authors of the article is complete (from the field <AuthorList complete>),
- the size of the abstract (from the fields <AbstractText> or <OtherAbstract> inside the field <Abstract> by counting the number of characters of the abstract),
- the keywords of the article (from the fields <Keyword> inside the field <KeywordList>),
- the title of the article (from the field <ArticleTitle>),
- the type of article (from the field <PublicationType>).

To identify articles published by editors between the start of their doctorate (PhD or MD) and the end of their research experience (occurring before they were recruited by *Nature* journals), we used their full first name and their full family name and compared them to the full first name and the full family name of each author of each article. The comparison addressed special characters (e.g. accents): if an author’s name matched the editor’s name after transforming any special character into the regular character from which it is originated (e.g. é, è ë, ê are all originated from e) the article was associated to the editor.

### Extraction of article data for Nature journals

We used the output of §1.6.1. Within each *Nature* journal and within each year of publication, we computed the number of original articles and the number of remaining articles. Within the original articles of each *Nature* journal, we computed for each year of publication the number of articles in which all authors were new (using the definition given in §1.5.1) and the number of articles with a known intermediate author. Therefore, for each original article of a given year n of publication, we looked whether each author of that article had published another original article within the two years before in one of the *Nature* journals included in the study. If that was not case for each author of the article, that article was counted as an article with only new authors. We did the same computations for known intermediate authors instead of new authors: an article was counted as an article by known intermediate authors if the first and the last authors were new authors and at least one of the other authors was known (i.e. not new).

We focused on known intermediate authors because publication bias favoring known first or last authors may be the consequence of last author’s better skills to shape the manuscript to the expectancies of *Nature* journals and/or of the Chaperone effect [16] instead of a bias in the publication process.

This extraction was repeated for the seven time windows specified in the definition of a new author (see §1.5.1).

### Extraction of article data for editors

We used the output of §1.6.1. Homonyms among editors were addressed by computing for each editor the number of his/her publications during the doctorate (PhD or MD). Missing data for start or end of the doctorate were replaced dates of the start of the doctorate (PhD or MD), of the end of the doctorate and of the end of the academic research experience by an adapted average value for editors for whom dates were missing (see §1.6.5).

Data associated to editors were:

- the number of articles, of any kind, in which at least one author had one of the editor’s affiliations when this latter was working as a researcher (including the doctorate),
- the number of original articles published by each author irrespective of the authorship position of the author on the article.

The first set of data required to compare authors’ affiliation with the editor’s former research affiliations. For this comparison, we replaced the collected editor’s affiliations by a subset of words extracted from their affiliation. This subset removed all words that were non specific to the editor’s affiliation (e.g. “University of Princeton” was replaced by “Princeton”). The rationale for using this subset of words in the affiliation was that the same author’s full affiliation may have been referenced in different ways across articles (e.g. the university of Princeton may be referenced as “University of Princeton” or “Princeton University”).

The second set of data allowed categorizing whether authors were a former co-author f the editor.

These two sets of data focused on articles published during the two years before the editor’s appointment on one side and on the other side, the two years after. Because *Nature* journals’ editorial process lasts several months between the date of submission of an article and the date of publication [17] and that this duration varies between submitted articles within a given journal without any quantification by *Nature* journals of these variations [17], we shifted the two years period by eight months according to the following figure:

2 years after shifted

2 years before shifted

Shift

Time

2 years after

2 years before

Editor’s arrival at Nature

The shift of eight months was designed to take into account the duration of the publication process so that the articles published during the two years after the editor’s arrival have been processed by the editor and not by the former editor. The duration of eight months has been computed by considering an average median duration in the editorial process across all *Nature* journals of six months [17] and two additional months of editorial training (supp. §2.4.4). We repeated the computations for shifts of six to ten months by steps of one month in order to assess the robustness of the results.

### Extraction of data for retracted articles

In each CSV file downloaded from the PubMed graphical user interface (see §1.4.4), we first extracted articles which constituted the acknowledgment of the retraction of an initial article. These acknowledgment articles had a title which started with “Retraction Note” or “Retracted Correction”. We collected the title and the date of publication of these articles. Their date was considered as the retraction date of the initial article. For each of these acknowledgment articles, we extracted the initial article by matching the titles. If the match was successful, the retraction and the initial article constituted a pair. We performed the same matching procedure by title for all remaining articles (i.e. the title of these articles did not start with “Retraction Note” nor “Retracted Correction”): if two of these articles had the same title but had distinct publication dates, the second article was considered as the retraction note of the first one. The most recent article was considered the retraction note while the oldest article was considered the initial article. All articles which could not be paired and which were not a retraction were considered initial articles. We extracted the authors and the publication date of all initial articles.

For each pair, we computed the duration of the retraction process as the difference between the date of publication of the retraction and the date of publication of the initial article. Note that we did not set any restriction over the article types (i.e. original articles, reviews…). The rationale was that the total number of retracted articles would have been too limited to allow a statistical analysis in subgroups of articles with enough statistical power.

### Handling of missing or incomplete data

Missing data involved editor’s years of start and end of the doctorate (PhD or MD), year of the end of the academic research experience and editors’ ORCID identifiers.

We computed the average duration of the doctorate and the duration of the research experience after the doctorate over all editors with available data. The average duration spent in academic research was computed as the sum of the two previous averages. The duration of the doctorate was computed as the difference between the year of the end of the doctorate and the year of the start of the doctorate. For the duration of the research experience after the doctorate, it was the difference between the year of the end of the research experience and the year of the end of the doctorate.

We then replaced missing values as follow: a missing year for the start of the doctorate of an editor was computed as the difference between the year of the end of the doctorate for that editor and the average duration of the doctorate computed as mentioned above. If the ending year of the doctorate was also missing for that editor, the same computation was performed except that we used the year of the end of the editor’s research experience and the average duration in research. Editors without any starting year of the doctorate, ending year of the doctorate and ending year in research could not be analyzed. They were discarded from the statistical analyses using the duration of the doctorate or the research experience after the doctorate. We followed the same procedure if the year of the end of the doctorate was missing or if the year of the end of the research experience was missing.

We did not use the editors’ ORCID identifier to address homonym issues as this information has been introduced very late (i.e. in 2012 [18]) and could not be used for editors who were appointed at *Nature* journals before.

Incomplete data involved authors’ affiliation in published articles. This case was encountered when at least an author lacks his/her affiliation. Two cases were taken into account: only one author in the article had an affiliation reported and at least two authors (but not all the authors of the article) had their affiliation reported but two of these affiliations were different.

In the first case, the affiliation of all authors was set to the affiliation reported. The rationale of this data assignment is that authors may have used a shortcut to report their affiliation: they set the affiliation of the first or last author and expected that readers would extrapolate it to the other authors.

In the second case, authors with no affiliation were ignored in any analysis involving affiliation. The rationale is that these missing data are likely related to a mistake of the authors or an error in the submission system rather than a rule of the authors.

Finally, there might have been cases in which several authors, but not all of the article, had their affiliation reported and their affiliation was the same. We addressed this case in the same way as the second case. The rationale is the same as for the second case.

### Handling of homonyms

#### Building groups of articles

This algorithm handled articles published by editors during their academic research career (i.e. from the start of the doctorate (PhD or MD) to the end of the last position in an academic research institution). These articles may have been published in any journal and were thus not restricted to *Nature* journals. For each editor, the algorithm grouped articles by shared characteristics that complemented the the editor’s authorship: another author, one of the affiliations of the editor collected from his/her biography and mentioned as the editor’s affiliation in the article (see §1.4.2), keywords… Thus, two articles who shared the name of one author in addition to the editor’s authorship were in the same group. Groups of article were merged each time one article of group 1 shared a characteristic with one article of group 2. The algorithm and a detailed description of its principles are available with the shared data of the experiment at https://entrepot.recherche.data.gouv.fr: <https://doi.org/10.57745/3QL466>.

This grouping aimed at refining the detection of homonyms of the editor among articles associated with the editor.

#### Identifying homonyms among editors and authors

Editors: Homonyms among editors may have biased the estimate of the number of articles published by editors as first and last authors during their experience of academic research before being recruited by *Nature* journals. It is the same for the estimate of the publications of their former co-authors and of authors affiliated to one of their former research institutions. Removing editors with homonyms is critical for all our analyses in which we used the number of articles published by editors as a regressor or an independent variable. The same consideration applied for the analyses of the number of articles published by editors’ former co-authors and authors affiliated to one of their former research institutions (figures 2A, 3B, 3C and supplementary figure 2A).

We used the number of articles of any type published by each editor during his/her doctorate (PhD or MD). This number was computed in the second step of the extraction of article data for editors (see §1.6.3). We computed the ratio of the number of articles published by the editor during his/her doctorate to the duration of his/her doctorate in years. We focused on the dissertation work rather than on the research period after the doctorate. It provided an independent way to assess the quality of the filtering. The number of publications expected from the doctorate period is more standardized than the number of publications after the doctorate. The size of the research team of an established researcher or the advisory work of advanced postdocs with PhD or MD students contributes to increase the number of publications of the researcher. However, during the doctorate, the researcher does not have such opportunities. These two parameters which modulate strongly the number of publication of a researcher after the doctorate are highly heterogeneous between researchers.

To identify editors who would have had homonyms, we ran the following algorithm starting with the 306 editors for whom we could compute this ratio:

*Loop until no more editor is removed:*

1. *Compute the average of the aforementioned ratio and its standard deviation (SD) over the set of available editors*
2. *Find all editors of the set whose ratio is strictly higher or strictly lower than the average + 2*SD*
3. *Remove these editors from the set*

The final set included 190 editors. They published a maximum of 1.25 articles per year of doctorate (PhD or MD). This maximum value seemed fairly reasonable: a student who manages to publish 5 articles over 4 years of doctorate may be considered as an excellent student. Editors who had published more than this threshold (i.e. 116 editors) were considered as having homonyms and thus discarded from the statistical analyses. We thus considered 190 editors in the statistical analyses (figure 1).

To check the robustness of this result, we reran the algorithm after replacing the bound of the exclusion range by 2.5*SD instead of 2*SD. 2.5*SD is a standard threshold used to remove outliers in a distribution, likewise 2*SD and 3*SD. Editors who would have been included in the final set would have published a maximum of 1.8 articles per year of doctorate. This maximum would have reached 2.8 if the bound had been equal to 3*SD. An average of 1.8 or 2.8 articles published per year during the editor’s doctorate was obviously unlikely suggesting the presence of homonyms among the set of editors who would have been included in the statistical analyses.

This method uses the number of articles published by the editor during his/her doctorate to identify homonyms among editors. It may not capture all editors who would have homonyms. Some editors may successfully go through this algorithm but elicit an important number of co-authors. This number may be highly heterogeneous between research fields and between types of experiments within a research field. For all analyses focusing on editors’ co-authors (see §1.9.3.1, §1.9.3.2 and §1.9.3.6), we thus complemented this method by removing all editors who had a total number of co-authors higher than *average+2*SD*. This led to remove 3 editors. The same editors were removed when using the threshold *average+2.5*SD* or *average+3*SD*. The use of the number of co-authors of the first group of co-authors identified with the algorithm of §1.6.6.1 led to remove only 2 of these 3 editors for the three thresholds *average+2*SD*, *average+2.5*SD* and *average+3*SD*. Since the third editor had inconsistent results, we decided to remove the three editors from all analyses focusing on editors’ co-authors. Of note is that we did not use the algorithm described at the beginning of this paragraph to identify these three editors because the distribution of the number of co-authors prevented the algorithm from converging.

Authors: likewise for editors, the computation of the number of original articles published by each author during the two years before the editor’s appointment and the two years after (see the third step in §1.6.3) may be biased by homonyms. We identified homonyms among these authors by removing authors who published more than *average+5*SD* articles over the four years (i.e. the two years before the editor’s appointment and the two years after). We considered all authors who published original articles in the editor’s journal. We also identified homonyms using the algorithm used for editors described above (in §1.6.6.2) with the total number of articles published by authors over the four years and the threshold *average+5*SD* instead of *average+2*SD*.

In the analysis reported in §1.9.3.1 (analysis of the publications of editors’ co-authors), this range led to exclude all authors who published strictly more than eleven original articles over the four years of interest (i.e. two years before the editor’s appointment and two years after). Note that these results are the same whatever if we consider all co-authors of the editor or only the first group of co-authors of the editor (see §1.6.6.1): the co-authors who are not in the first group of the co-authors of the editor are considered as non co-authors when running the analysis with the first group of co-authors.

Authors who have published between one and 11 articles over the four years were thus included in the analysis. A more strict inclusion range (i.e. *average+3*SD*) would have led to exclude all authors who published strictly more than seven original articles over the four years: this threshold would have likely excluded wrongly the subset of the most prolific authors. Note that the number of articles published by an author does not discriminate the authorship position. Therefore, even if an author publishes two articles as first or last author in a *Nature* journal over four years, his/her collaborations may lead him/her to publish substantially more than these two articles. On the opposite, extending the inclusion range to *average+7*SD* would have led to exclude all authors who published strictly more than 15 original articles over the four years of interest : this would have represented the exclusion, in the analyses, of 0.2% of the data which had to be compared to the exclusion of 0.25% of the data for the inclusion range *average+6*SD*, 0.34% for *average+5*SD*, 0.48% for *average+4*SD* and 0.74% for the inclusion range *average+3*SD*.

The use of the algorithm with the range *average+5*SD* in the analysis reported in §1.9.3.1 led to exclude all authors who published strictly more than seven original articles over the four years of interest (i.e. two years before the editor’s appointment). A more strict inclusion range (i.e. *average+3*SD*) would have led to exclude all authors who published strictly more than three original articles over the four years: this threshold would have likely excluded wrongly the subset of the most prolific authors. Note that the number of articles published by an author does not discriminate the authorship position. Therefore, even if an author publishes one article as first or last author in a *Nature* journal over four years, his/her collaborations may lead him/her to publish substantially more in this journal. On the opposite, extending the inclusion range to *average+7*SD* would have led to exclude all authors who published strictly more than thirteen original articles over the four years of interest: this would have represented the exclusion, in the analyses, of 0.99% of the data which had to be compared to the exclusion of 1.39% of the data for the inclusion range *average+6*SD*, 2.12% for *average+5*SD*, 3.63% for *average+4*SD* and 7.53% for the inclusion range *average+3*SD*.

The seven fold ratio between the rate of excluded data with a threshold of *average+3*SD* and *average+7*SD* and the substantial proportion of removed data for the threshold of *average+3SD* shows that the amount of homonyms among authors publishing in the same journal need to be assessed and addressed to justify an absence of biases in the results contrary to a speculation arguing for a limited number of homonyms as used previously [16].

#### Dates

Because dates of recruitment of editors were not fully available with a precision of a day, we defined an exclusion period around the date based on its precision: if the month (but not the day in the month) of the date was available, any article published during that month was excluded from analyses. If the season was available (but not the month), any article published during the season, defined with a precision of a month, was excluded from the analyses (i.e. winter=January to March included, spring=April to June included, summer=July to September included, autumn=October to December included). If a semester was available, any article published during that semester, defined with a precision of a month, was excluded from the analyses (i.e. first semester =January to June included; second semester: July to December included). The rationale of this exclusion period was that it was impossible to know whether an article published during the exclusion period was published before or after the editor’s appointment: in the case of an editor who joined *Nature* journals in March, it was impossible to know whether an article published the 15^th^ of March had been published before or after the arrival of the editor at *Nature* journals. The reasoning remains after considering a shift in time of 8 months between the editor’s appointment and the date of publication of an article to take into account the duration of the publication process and the training of the editor (see the third step in §1.6.3).

### Homonyms among authors of articles published in Nature journals

Homonyms among authors of articles of *Nature* journals would have reduced the rates of articles published by new authors each year in each *Nature* journal. We thus considered that authors whose name was found in more than 50 articles published over the entire duration of publication of a given *Nature* journal were new authors for each of these articles. Note that this rule is in favor of *Nature* journals. Nonetheless, we ran a robustness analysis without this threshold to check that we obtained consistent results (see §1.11). We could not run the same algorithm as in §1.6.6.1 because the computational time would have required 6 months per author consistently with previous data on computerized name disambiguation [19].

### Year of launch of Nature and Science journals

For each journal, we used the earliest year between

- the year of launch of the journal collected according to §1.4.1,
- and the year of the oldest article published in the journal and referenced in PubMed.

### Handling of programming errors

All programs used for data extraction included a substantial number of functions and programming lines. This might have been a source of error. Automated data quality checks were programmed and ran at the end of each step of the data extraction (see §1.6.1, §1.6.2 and §1.6.3). Additionally, all these programs were reviewed manually three months after the end of the analyses to reduce the risk of errors as recommended [20]. Whenever an error was detected, the programs were corrected if the error could have impacted the results, additional data quality checks were performed and analyses were rerun.

A second revision and additional data quality checks were performed with the number of co-authors one year later for the extraction of articles published by editors’ former co-authors in *Nature* journals. Whenever an error was detected, the programs were corrected if the error could have impacted the results, data quality checks were performed and the analyses were rerun.

### Duration of the periods before and after the editors’ appointment used to investigate conflicts of interests

In the results related to the potential conflicts of interests between editor and authors (see §1.9.3.1, §1.9.3.2, §1.9.3.3, §1.9.3.6 and §1.9.3.7), we used a duration of two years to define the periods before and after the editors’ recruitment by his/her current *Nature* journal. This duration was set to 1, 3, 4, 5, 10 years and the entire-range of publication of the journal in robustness analyses. These analyses aimed at checking that the choice of 2 years did not bias the results. Note that for analyses of §1.9.3.6 and §1.9.3.7, we could not run these robustness analyses because the computational time to extract the data would have been around six months for each editor.

However, these two periods (i.e. before and after the editor’s appointment) had to be truncated when the recruitment date of the editor was too close to the launch date of the journal or too close to the end date of data available in the PubMed database (i.e. December 12 2020). For each of these editors, without this truncation, there would have been an unbalanced dataset between the periods before and after the recruitment of the editor (the two figures below are made for a duration of two years of the two periods):

Truncated period after

Time with no article for the editor’s journal

Truncated period before

Time

Editor’s appointment

Launch of the editor’s journal

2 years before

2 years after

And for the end date available in the PubMed database:

Truncated period after

Time with no article for the editor’s journal

Truncated period before

Time

2 years after

December 12 2020

Editor’s appointment

2 years before

The unbalanced dataset would have been the consequence of the time window during which there was no article published in the journal. Therefore, in these cases, we truncated the duration to the minimum duration between the two values:

- the duration in months between editor’s recruitment and launch date of the journal
- the duration in months between the end date of available data in the PubMed database and the editor’s recruitment

This truncation was performed only for analyses reported in §1.9.3.1, §1.9.3.2, §1.9.3.3, §1.9.3.6 and §1.9.3.7. All editors for whom we truncated data were still included in the analysis with the truncated duration set for the two periods. This inclusion favors by construct an exponential shape in the robustness analyses when the duration defining the two periods increases (see §1.11). However, because this truncation occurs more likely for the longest durations used to define the two periods, the expected exponential shape confirms rather than ruins the reported results when the reported results are still significant for the longest durations used to define the two periods.

## Data analyzed

### Editors

Only editors who made decisions about submitted primary research reports (i.e. original articles) and comments on such reports were included in the analyses. Among those editors, were discarded from the analyses:

- those whose arrival date in their current journal was not available or too late with a too imprecise date to allow a period of publication after the exclusion period (n=16, figure 1, e.g. an editor whose appointment date was 2020 without any month or season mentioned had an exclusion period over 2020 (see §1.4.2.1) making thus the analysis of articles published after the editor’s appointment impossible since the PubMed database had no article after December 2020),
- those who had homonyms (n=115, figure 1),
- and those who were outliers for the number of articles published (n=1).

Outliers among editors were defined as editors who had a number of original articles published as first author before leaving academic research higher than the average+2*SD. We thus considered 190 editors for the statistical analyses. Editors who had homonyms or who were outliers were used to assess the robustness of the results (see §1.11).

Some editors (2.2%, n=7) worked simultaneously in the editorial boards of several journals included in the study. Those editors who survived the aforementioned three exclusion criteria were included in the analyses as many times as they worked for a distinct journal because their editorial decisions may differ between journals. This approach may overweigh the role of these editors as compared to the other editors. However, the analyses with the lower number of editors which would be the most sensitive to this overweigh (i.e. §1.9.3.1 and §1.9.3.6) had additional exclusion criteria which discarded all these 7 editors.

### Journals

Only *Nature* journals which published articles before December 12 2020 were included in analyses which addressed questions related to published articles (n=31 journals). In all other analyses, all *Nature* journals included in the study were considered (n=33 journals).

### Articles

Out of the 31850051 articles referenced in the PubMed database on December 12 2020, we extracted 98002 original articles out of a total of 210205 articles published in the *Nature* journals included in the study.

Finally, we retrieved 313 retracted articles and retraction notes referenced in PubMed which were published after January 1^st^ 1990 in the *Nature* journals included in the study. Out of these 313 articles, 129 were labeled as retraction notes or retracted corrections and 126 had a title and could thus be potentially paired with an initial article having the same title. A total of 116 articles were paired with this approach. We also paired 16 other articles whose titles were the same but their publication dates were different and these articles were not labeled as a retraction note or a retracted correction. We therefore considered 132 pairs of articles for the analyses of retracted articles. Within each pair, the oldest article was considered as the initial article and the latest one, the retraction of the initial article.

### Job offers

From December 2020 to December 2021, we collected 187 job offers for editorial positions at journals which are part of the *Springer Nature* group out of which 107 were for editorial positions in the *Nature* journals included in the study and meeting inclusion criteria. 29 were offers advertised several times leaving 78 job offers for analyses. Of note were eight additional job offers for such editorial positions aiming at making decisions about submitted manuscripts for new *Nature* journals to be launched in 2022 and 2023 and publishing primary research reports: *Nature Water* (2023), *Nature Mental Health* (2023), *Nature Cardiovascular Research* (2022) and *Nature Synthesis* (2022).

## Material

All computations were made with Matlab r2017a and r2019b (IN2P3 computing grid). General linear models (GLM) used the anovan and robustfit (for robustness analyses) functions of Matlab, and linear mixed models were run with the fitlme and fitglme functions of the Matlab statistical toolbox for models involving large number of data (e.g. over 6 million data for analyses of §1.9.3.1) and, for the others, R 3.5.1 using the lmerTest package. The REML (Restricted Estimate of the Maximum Likelihood) method was set to estimate the models with the fitlme function to match the lmerTest function. The fit method for the fitglme function of Matlab was set to Laplace. All data extractions were performed with parallel computing using Linux and qsub job management on a computing grid of the IN2P3.

## Statistical analyses

### Computations

#### Quantification of the scientific and editorial experiences of editors

First, using the dataset of editors’ co-authors computed in the second step of §1.6.3, we computed, for each editor, the number of editors’ original articles published as first or last author between the start of their doctorate (PhD or MD) and the end of their experience in research. The rationale for taking into account only articles published as first author or last author was that first authors are researchers who are the most involved in the study and last authors are researchers who supervise the work [21]: thus these articles better capture the editor’s skills that are expected from a researcher than articles published as intermediate or middle authors [21]. We considered the 190 editors included in the analyses (see §1.6.6). This computation characterized a part of the editorial board’s scientific experience.

Second, considering the same 190 editors, we computed, for each journal, the average duration of the research experience of editors. The duration of the research experience of an editor was defined as the difference in years between the end of his/her research experience and the start of his/her doctorate. This second computation characterized another component of the editorial board’s scientific experience: the time spent in research and the number of original articles published as first or last author by editors during their doctorate correlated only moderately (r_Pearson_=0.37, p<0.0001).

Third, considering the same 190 editors, we computed for each editor the duration (in years) as editor at *Nature* journals of its current editorial board. This duration was computed for each editor as the difference in years between December 2020 and the year of appointment at *Nature* journals as editor. This duration characterized the editorial experience of the editor at *Nature* journals. Note that, for a given editor, the appointment as editor at *Nature* journals may have been in a *Nature* journal distinct from the current journal where the editor works.

#### Nature journals

*Nature* journals were classified in generalist journals (i.e. *Nature* and *Nature Communications*) and specialized journals (i.e. all other *Nature* journals). The rationale of these two classes was that editorial board behaviors may have been different between general and specialized journals due to the difference in readership.

The year of the first original article published by each *Nature* journal was computed from the dataset of articles published in *Nature* journals coming from the output of the first step of the extraction of article data for *Nature* journals (see §1.6.1). This consideration did not apply to *Nature* whose launch date was 1869 [22] and for which the PubMed database referenced only very partially the articles published before 1990 (see §1.5.2). We set the start of *Nature* in 1990 which was two years before the launch of the first *Nature* journal, *Nature Genetics*. This allowed to have a consistent weighting of the time series of data between *Nature* journals. Otherwise *Nature* would have over-weighted the fits of the linear models.

### General statistical considerations

All linear models with the regressors of the editors’ scientific (i.e. time spent in academic research and number of original articles published as first or last author) and editorial experiences (i.e. time spent as editor at *Nature* journals) included, whenever relevant, these three regressors systematically in order to have a consistent modeling approach and to improve the reliability of the results.

In all linear mixed models, we used the Satterthwaite approximation to compute the degree of freedom in order to get the p-value of the t-statistic applied to the coefficients of the model.

All linear models using time series of the number of articles published by *Nature* journals (or percentages of them) included time series tailored to the duration of the publication of each journal. For instance, the time series of the number of original articles published each year for *Nature Neuroscience* ranged from 1998 to 2020 while the same time series for *Nature Methods* ranged from 2004 to 2020 leading thus to a lower number of time points for *Nature Methods* than for *Nature Neuroscience*. The unequal number of time points between *Nature* journals was addressed with mixed models. We used percentages of original articles with new authors or percentages of retracted articles as the dependent variable rather than a binary one in models because we addressed issues related to journals’ editorial behaviors.

The Bonferroni corrections for multiple testing were used whenever appropriate. The reported standard errors are not clustered standard errors.

### Statistical models and tests

Statistical models and tests reported in this paragraph are the ones that led to the results reported in the main text. The order of reporting of these models is the same as the one of the reporting of their results in the main text. Any statement in the main text referring to supplementary analyses refers to models that are reported in the supplementary analyses section (see supp. §1.10).

#### Analysis of the number of articles published by editors’ former co-authors and the other authors in the editor’s journal

We compared the 2-year period before the editor’s appointment to the 2 years after. We discriminated the editor’s former co-authors from the other authors. We focused only on articles published in the editor’s journal. Among the 190 editors included in the analyses (figure 1), we considered only editors for whom there was at least a co-author identified (n=156). Among these 156 editors, 3 were excluded because of too many co-authors (see §1.6.6.2). We included only editors (n=56 editors out of the 153 remaining) who had at least a co-author who published at least an article during the two years before the editor’s appointment at *Nature* journals or during the two years after. The rationale for excluding the other editors from the analysis is that it is impossible to know whether the absence of publication of a co-author in the editor’s journal is the consequence of an absence of submission to that journal or a rejection. An absence of submission cannot be related to editors. Note also that the appointment at *Nature* journals may have occurred before the date the editor joined his/her current journal. Editors in this case were included in the study if the first *Nature* journal they worked for was one of the journals included in the study. The rationale is that a reputational bias in favor of former co-authors is more likely to occur when editors start their editorial career than after several years.

For each of these 56 editors, the median number of authors over the four years (i.e. the two years before the editor’s appointment and the two years after) was 56850 (interquartile: [5775 – 130417]. These authors contributed to at least an article which was published either during the two years before the editor’s appointment or the two years after. The large number of authors is due to editors working for *Nature Communications*. Among these authors, 4.3 authors on average (SD=3.8 authors) were former co-authors of the editor. These authors may have homonyms. Those with homonyms were removed from the analysis (see below). Note that we identified for editors who had an editorial experience in previous *Nature* journals the first *Nature* journal for which the editor worked and which was included in the study. Editors who had a first editorial experience in a *Nature* journal which was not included in the study (e.g. *Nature Review* journals) were excluded from this analysis.

We used the output of the third step of §1.6.3. For each author who published in the editor’s journal and for each period (i.e. the two years before the editor’s appointment and the two years after), we computed the total number of articles published in the editor’s journal. Thus each author, former co-author of the editor or not, who had published at least an article in the editor’s journal during the two years before the editor’s recruitment or the two years after had two numbers:

1. The total number of articles published in the editor’s journal before the editor’s appointment
2. The total number of articles published in the editor’s journal after the editor’s appointment.

Note that one of these two numbers may be equal to 0 but not all of them. We removed all authors who had homonyms by considering the total number of articles published in the editor’s journal over the two periods (see §1.6.6.2).

We then ran a linear mixed model. The difference between these two numbers was modeled as a linear function of the type of author (editor’s former co-author/other author), the number of articles published by each author before the editor’s appointment (i.e. first number aforementioned) and editors as a random effect over the intercept. The regressor with the number of articles published by each author allows to adjust the regressor of interest (i.e. the type of author) over the publication record of each author in the editor’s journal. The rationale is that there were differences between former co-authors of the editor and the other authors in the number of articles they published in the editor’s journal before the editor’s appointment (see §1.10.8). Additionally, authors with a higher track record in *Nature* do not seem to have the same chances to publish subsequently in *Nature [16]*.

To test whether the difference between the types of authors was associated with the editor’s scientific experience, we ran a linear mixed model. The difference in publications between after the editor’s appointment and before was modeled as a linear function of:

1. the type of author,
2. the duration spent in academic research by the editor,
3. the interaction of regressor 2 with the type of author,
4. the number of original articles published as first or last author by the editor during his/her experience in academic research,
5. the interaction of regressor 4 with the type of author,

Editors were set as random factors over the intercept. The interactions of regressors 1 and 3 with the type of author allowed to test whether the difference in the increase in publications between editor’s former co-authors and the other authors was associated with the scientific experience of the editor.

The two time windows of two years (i.e. before and after the editor’s appointment) were locked over the eighth month after the arrival of the editor at *Nature* journals (see the figure in the third step of §1.6.3): six months accounted for the average, over all analyzed *Nature* journals, of the median duration of the editorial process of a submitted manuscript to its final editorial decision inside each journal [17] and two months accounted for the duration for publishing the article (i.e. from the final editorial decision to the publication date) and the training of the editor mentioned in job offers for associate or senior editor positions at *Nature* journals (see §2.4.4).

To test the robustness of the results of the first model described in this paragraph (which results are reported in figure 2A), we ran it again with the thresholds *average+3*SD*, *average+4*SD*, *average+6SD* and *average+7SD* used to identify homonyms among authors (see §1.6.6.2), with the algorithm used to identify homonyms for each of these thresholds (see §1.6.6.2) and with the first group of editor’s co-authors (see §1.6.6.1) with each of these two methods to remove homonyms. Note that the first group of co-authors of the editor may be too restrictive: it may not include co-authors of the editor when this latter has moved from a research institution to another without keeping ties with the former research institution. On the contrary, the full set of co-authors may be the consequence of editors’ homonyms after the end of his/her doctorate. Therefore the results of these robustness analyses represent lower and higher bounds. Significant results using both the first group of editor’s co-authors and the full set of co-authors suggest that homonyms do not account for our results.

However, we did not test the robustness of the results with regards of the duration of the time window used to define the period before and after the editor’s recruitment by his/her current journal (i.e. we used in the other robustness analyses durations of 1, 3, 4, 5, 10 years instead of 2 years and the entire duration of publication of the journal) nor did we assess the robustness of the results with regards of the duration of the shift (i.e. 6, 7, 9 and 10 months instead of 8 months) used to take into account the duration of the publication process and the training of the editors. The rationale to not run these robustness analyses was that the computation time to extract the data would have been around six months for each editor.

#### Quantification of chances to publish in the editor’s journal as a function of the author’s publishing record (or experience in publishing) in this journal and the author’s status to the editor (i.e. former co-authors vs other authors)

We included in this analysis only authors who published at least one article in the editor’s journal in the two years before the editor’s appointment. Authors were either editor’s former co-authors or other authors. The obtained dataset is the same as the one of the analysis reported in §1.9.3.1 except that all authors who had no article published during the two years before the editor’s appointment were removed. Then, we set a binary variable for each author to capture whether the author published during the two years after the editor’s appointment. The number of publications before the editor’s appointment captures the author’s experience in publishing in the editor’s journal. This view of the number of articles published before the editor’s appointment is substantially different from the analysis of §1.9.3.4. In §1.9.3.4, articles considered were published after the editor’s appointment. Editors had therefore the opportunity to have heard from these authors before making a decision. It could have been through the regular editorial meetings where editors discuss submitted manuscripts. However, in the current analysis, authors of articles published before the editor’s appointment have lower chances to have come to the editor’s attention.

We removed homonyms among authors in the same was as in §1.9.3.1 to get a comparable dataset for the analysis.

We ran a descending logistic regression with mixed effects. We used the fitglme function of Matlab. We modeled whether authors published after the editor’s appointment as a logit function of:

1. the number of articles published by authors during the two years before the editor’s appointment,
2. the type of author (editor’s co-author/other author),
3. the interaction between regressors 1 and 2

We set the editor as a random factor over the intercept. Other authors are authors who are not former co-authors of the editor. The interaction between regressors 1 and 2 captures whether the influence of author’s experience before the editor’s appointment over his/her chances to publish after the editor’s appointment differed between editors’ co-authors and the other authors. Whenever, this effect was not significant, regressor 3 was removed and the model re-estimated (i.e. descending procedure in a general linear model).

To test the robustness of the results of this model, we ran it again with the thresholds *average+3*SD*, *average+4*SD*, *average+6SD* and *average+7SD* used to identify homonyms among authors (see §1.6.6.2), with the algorithm used to identify homonyms for each of these thresholds (see §1.6.6.2) and with the first group of editor’s co-authors (see §1.6.6.1) with each of these two methods to remove homonyms likewise in §1.9.3.1. Additionally, and for the same reason as in §1.9.3.1, we did not test the robustness of the results with regard to the duration of the time window used to define the period before and after the editor’s recruitment by his/her current journal (i.e. we used in the other robustness analyses durations of 1, 3, 4, 5, 10 years instead of 2 years and the entire duration of publication of the journal). For the same reason, we did not assess the robustness of the results with regard of the duration of the shift (i.e. 6, 7, 9 and 10 months instead of 8 months) used to take into account the duration of the publication process and the training of the editors.

#### Analysis of the total number of articles published by authors with the same affiliations as the editor when he/she was working as a researcher

We compared the 2-year period before the editor’s appointment to the 2 years after. We discriminated the editor’s journal from the other *Nature* journals. We focused only on articles published by authors affiliated to institutions where the editors had worked as a researcher before their appointment at *Nature* journals. Likewise in §1.9.3.1, we included only editors for whom at least one author with one of the editor’s former academic research affiliations published at least one article in one of the other *Nature* journals (i.e. not the one of the editor) during the two years before the editor’s appointment at *Nature* journals or during the two years after (n=111 editors out of the 190). Note that the appointment at *Nature* journals may have occurred before the date the editor joined his/her current journal. Editors in this case were included in the study if the first *Nature* journal they worked for was one of the journals included in the study.

We used the output of the third step of the extraction of article data for editors (see §1.6.3). For each editor, we computed the total number of articles published in each of the *Nature* journals included in the study by authors affiliated to one of the former editor’s affiliations. For each editor, we averaged these numbers across *Nature* journals except the editor’s one and *Nature* journals in which there was no article published by authors affiliated to one of the former editor’s affiliations during the two years before the editor’s appointment and the two years after. The rationale for excluding these journals from the average is that it is impossible to know whether the absence of publication of a given author in a given journal is the consequence of an absence of submission to that journal or a rejection. An absence of submission cannot be related to editors.

To remove the contributions of outliers, we removed the data associated to editors for whom the difference, between before and after the editor’s appointment, in the total number of articles with at least an author affiliated to one of the editor’s former research institution was strictly higher than *average+3*SD*. The handling of outliers is different in this analysis than in the ones of §1.9.3.1 and §1.9.3.2. The rationale is that affiliation data may have a lower quality than the one of authors: all authors’ names are available while the affiliation of each author may not be fully given (see §1.6.5). The method of §1.6.3 to classify accurately the affiliation of a given author reported in different ways across articles (e.g. “Princeton University” in an article and “University of Princeton” in another article) may also have not been systematically successful. These two effects would have likely introduced a high variability in the number of articles of each author.

Using a paired t-test, we compared the total number of articles published in the editor’s journal by authors with one of the academic research affiliations of the editor (when he/she was working as a researcher) over the two years before the editor joined *Nature* journals and the two years after. Using a paired t-test, we compared the averaged total number of articles published in the other *Nature* journals by authors with one of the academic research affiliations of the editor (when the editor was working as a researcher) over the two years before the editor joined *Nature* journals and the two years after. Using a paired t-test, we compared the two aforementioned differences. Since we performed three paired t-test, the Bonferroni correction was applied. We included in the analysis only the editors who did not have any previous experience as editor in another journal whose name started by *Nature*. The rationale of excluding these editors is that editors are more likely to have biased decisions at the beginning of their editorial career than after several experiences in the same journal family. Results of figure 2B support this assumption.

The two time windows of two years (i.e. before and after the editor’s appointment) were locked on the eighth month after the arrival of the editor at his/her journal (see the third step in §1.6.3).

The editors’ characteristics (i.e. time spent by the editor in academic research, time spent as editor at *Nature* journals, number of original articles published as first or last author during the academic research career) could not be used in this analysis to test whether the magnitude of the effect would be related to the editors’ experience. Indeed, the longer an editor has worked as a researcher, the more likely he/she has moved from an academic research institution to another.

This analysis is different from the one used for the editor’s co-authors. We used the total number of articles published by all authors affiliated to a former research institution of the editor. We also used a direct t-test. The rationale for this difference is that ties with the editors may be stronger for their former co-authors than for authors affiliated to one of their former research institution but with whom they have never interacted. Authors with the same affiliation as the former editor’s one may also work in different disciplines than the one of the editor. These authors may therefore submit more easily in other journals than the editor’s one contrary to editor’s former co-authors who work in the same discipline as the editor. Additionally, congruent results between two different statistical method used in two independent analyses testing two closely related questions (e.g. analyses of §1.9.2 and this analysis) suggest that any increase in publications after the editor’s appointment as compared to before is robust with regard to the choice of the statistical method.

To test the robustness of the results of this analysis, we ran them again with a time window locked over the 6^th^, 7^th^, 9^th^ and 10^th^ month instead of the 8^th^ month after the editor’ appointment. We also tested the robustness of the results by setting the duration of the time window to 1, 3, 4, 5, 10 years instead of two years and to the duration between the launch of the journal and the editor’s appointment (see §1.6.10).

#### Analysis of the dynamics of the articles published by new authors and known intermediate authors

Result 1: the percentage of original articles published by only new authors in *Nature* journals decreased over years while it was the opposite for articles by known authors with middle authorship position.

Using the output of the third step of the extraction of article data for *Nature* journals (see §1.6.2), we computed, for each year since 1990 included, the percentage of original articles published by only new authors within each *Nature* journal. Then we ran a general linear mixed model using all *Nature* journals. We modeled this rate as a linear function of time (locked on the launch of the *Nature* journal (i.e. time=1 for the first year of publication of the journal, time=2 for the second year of publication of the journal…)), the *Nature* journal type (i.e. generalist/specialized) and the interaction between time and the *Nature* journal type (table 2). Journals were set as a random factor over the intercept (table 2).

We included in the analysis only *Nature* journals which had at least three years of publication (n=25 journals) because the linear fit over time for one journal requires at least two points and the variability of the data would have substantially biased the fit in the case of journals with only two data points.

We ran the same computations and the same model with articles published by known middle authors instead of only new authors (table 2).

Result 2: the scientific and editorial experience of editors modulated the temporal dynamic of acceptation of articles with only new authors but not of articles with known middle authors.

We used the yearly rate of original articles published by only new authors and the yearly rate of original articles published by known middle authors computed for result 1 for each *Nature* journal. Then, within each *Nature* journal, for each editor of the journal, we extracted the rates computed for the years after the editor’s appointment at the journal. For instance if an editor was appointed at *Nature Methods* in 2017, yearly rates computed for *Nature Methods* were extracted for 2018, 2019 and 2020 for that editor while if another editor was appointed in 2012, yearly rates computed for *Nature Methods* were extracted for each year from 2013 to 2020 included for that editor. We did not include the year of appointment of the editor at *Nature* journals for the same reasons that we set an exclusion period around the arrival date of editors (see §1.6.6.3). This computation tailored to each editor allowed to take into account the differences in the time spent as editor in the journal between the editor’s appointment at the journal and 2020. Finally, we ran a linear mixed model (table 2). We modeled the rate of original articles published by only new authors (the rate is tailored to each editor as aforementioned) as a linear function of time (locked on 2020), the *Nature* journal type (i.e. generalist/specialized), the number of articles published by the editor as first and last author during his/her academic career, the time previously spent by the editor in research institutions starting at the year of the start of the doctorate, the time spent as editor at *Nature* journals, the interactions between time and the *Nature* journal type (i.e. generalist/specialized) and the interaction between time and each of these three regressors separately. Editors were set as a random factor over the intercept. The interaction between time and the number of original articles published by the editor as first or last author and the interaction between time and the duration spent in research by the editor captured the association between the scientific experience of editors and the evolution across time of the publications of original articles with only new authors in *Nature* journals. Similarly, the interaction between time and the duration spent as editor at *Nature* journals captured the association between the editorial experience of editors and the evolution across time of the publications of original articles with only new authors in *Nature* journals. The duration spent as editor at Nature journals may also be interpreted in this analysis as a quantification of a bias related to a bias of selection of the editors (see §1.10.9). However, other analyses targeting periods of time before the editor’s appointment or shortly after it (i.e. the two years after the appointment) suggested that there was little chance of a bias related to the selection of editors (see §2.4.6, §2.4.7 and §2.4.8).

We included in the analysis only editors of *Nature* journals which had at least three years of publication (n=25 journals) because the linear fit over time for one journal requires at least two points and the variability of the data would have substantially biased the fit in the case of journals with only two data points. This consideration is identical to the one of result 1.

The same linear mixed model was carried out with the rate of original articles of known middle authors (table 2).

#### Association between the journal’s impact factor and differences in publications between new authors and established authors

General considerations: We included in the analyses of the results reported below only *Nature* journals which had at least three years of publication (n=25 journals) because we computed the difference of the main variable of the analysis between two consecutive years for two of these results (results 1 and 2) and we did a linear fit over time of this difference (result 3) which requires at least two points.

Result 1 (figure 3A): the number of original articles by new first and last authors in *Nature* and *Nature Communications* decreased the year after a decrease of the IF of the journal and vice-versa. This decrease was stronger than for articles in which the first or the last author had already published in *Nature* journals before.

Using the output of the third step of the extraction of article data for *Nature* journals (see §1.6.2), we computed, for each journal, the difference in the number of original articles by new first and last authors between year n+1 and year n. We also computed the difference in the IF of the journal between year n and year n-1. We did the same for all other original articles in each journal. We then ran a linear mixed model in which the difference in the number of original articles was modeled as a linear function of the difference in the IF, the type of articles considered (i.e. articles by new first and last authors/other original articles), the type of journal (generalist/specialized), all two-way interactions and the three-way interaction. The journals were set as a random factor over the intercept. The interaction between the type of articles and the IF captured a difference, between new and established authors, in the association between the variations in IF at a given year and the variations one year later in the number of articles published by these authors. We focused on the results for generalist journals because of the steeper slope between the rate of articles with new first and last authors at year n-2 and IF at year n in generalist journals as compared to specialized journals (see §1.10.3 and §2.4.2): a steeper slope suggests a stronger sensitivity of IF to articles of new first and last authors justifying a stronger motivation to regulate it.

One may object that a difference in the number of original articles between year n+1 and year n cannot be influenced by a difference in the IF between year n and year n-1 because of the duration of the publication process and because the IF at year n is released in June of year n+1. However, the final decision of acceptance of a manuscript is made by the editors shortly before the publication of the article: thus only the duration of the publication process after the final editorial decision can influence the results and this duration is extremely limited as compared to the six months on average of the duration of the peer review occurring before this final editorial decision (see the third step in §1.6.3). Additionally, it is possible to track (with *Google scholar* for instance) every day the number of citations of articles without waiting for the release of the IF. Finally, result 2 below takes such a potential bias already into account (i.e. the model focuses on the difference between year n+2 and year n+1 instead of year n+1 and year n).

Result 2 (figure 3A): the number of original articles by new first and last authors in *Nature* and *Nature Communications* decreased at year n+2 as compared to year n+1 when the IF of the journal decreased at year n as compared to year n-1 and vice versa.

We ran the same analysis as for result 1 except that all differences in the number of original articles were computed between year n+2 and year n+1 instead of year n+1 and year n.

Result 3: the relationship between the variations of the number of original articles of new first and last authors (year n+1 – year n) and the variations of the IF between year n and year n-1 (year n – year n-1) increased across years while it was not the case for articles with a known first or last author.

We ran the same analysis as for result 1 except that we added a time regressor in the model of result 1, all two-way interactions, all three-way interactions and the four-way interaction with the three regressors of the model of result 1. The time regressor was the difference between the year of publication considered (year n) and 2019: we did not included year 2020 because the 2020 IF was not available when we ran the analyses. We computed such differences to get an intercept of the model for the same year (i.e. 2019) for all journals. This difference was always negative: for example, if a journal had 8 years of publication until 2019, there were 4 values included in the model for that journal and the time regressor was:

- -1 when year n was 2018,
- -2 when year n was 2017,
- -3 when year n was 2016,
- and -4 when year n was 2015.

2014, 2013 and 2012 could not have been included in the analyses because, for each year n considered in the anlaysis, it required to use data of year n+1 and year n-1 and the IF was not available, by construct, during the first two years of publication of the journal.

Result 4: the variations of the number of submissions of original articles in *Nature* (year n+1 – year n) were independent of the variations of the IF between year n and year n-1 (i.e. the difference: year n – year n-1) while the number of published articles included in the computation of the IF was linearly associated with the variations of the IF between year n and year n-1 (i.e. the difference year n – year n-1).

We used the history of the number of submissions of original articles referenced on the *Nature* website, the history of the IF provided by Clarivate and the history of the number of published articles used to compute the IF and provided by Clarivate. These three histories ranged from 1997 to 2017. Of note is that the articles considered for the computation of the IF include original articles and exclude editorials, letters and meeting abstracts [23]. Editors are aware of how the IF is computed and which kind of article is considered for its computation [24]. Because the number of articles submitted is around 10 times larger than the number of published articles included in the computation of the IF [12], we normalized our data. We thus computed for each year n:

1. the difference between year n+1 and year n in the number of submissions divided by the number of submissions for year n
2. the same but with the number of published articles included in the computation of the IF
3. the difference between year n and year n-1 in the IF.

We then ran a general linear model in which we modeled the first two computations as a linear function of the third computation, the type of the first two computations (normalized difference in submissions/normalized difference in published articles) and their interaction. We used the glm function of R. Of note is that this model is a mirror of the model of result 1. It allows to test, with data publicly available, the contributions of the submission behavior of authors and of the editorial process in the results.

#### Comparison of changes in the number of publications by editors’ co-authors and the other authors before and after the setup of the non-financial conflicts of interest policy at Nature journals

This analysis is very close to the one of §1.9.3.1. Its difference lies in an additional regressor capturing whether the editor was appointed before or after the setup of the non-financial conflict of interest policy by *Nature* journals.

We excluded from the data those editors who were appointed between May 2015 and May 2017. The rationale is that these editors would have handled, during the two years following their appointment, manuscripts that would have been published, for some of them, before the setup of the non-financial conflict of interest policy and, for the others, after the setup of this policy. The 8 months shift between February 2018 and May 2017 takes into account the duration of the publication process and the duration of the training of the editor (see the third step in §1.6.3). Articles published within the 8 months after the editor’s appointment have been handled by other editors.

We also excluded editors who were appointed before September 16^th^ 2011. The goal was to have the same duration of the periods over which the editors were recruited before and after the setup of the non-financial conflict of interest policy. We therefore had 3.62 years between May 1^st^ 2017 and December 20^th^ 2020. It corresponded to the period during which editors have been recruited and have handled manuscripts which have been published after the setup of the non-financial conflict of interest policy. We also had 3.62 years between September 16^th^ 2011 and May 1^st^ 2015. The period between September 16^th^ 2011 and May 1^st^ 2015 included editors who handled only manuscripts that have been published before the setup of the non-financial COI policy. 37 editors were thus included in this analysis. These editors are a subset of the 56 editors of the analysis of §1.9.3.1. 44 editors were included in the analysis when considering editors who were appointed before September 16^th^ 2011 in the robustness analyses (see §1.11 and §2.3).

Likewise in §1.9.3.1, we removed homonyms among authors by using a fixed threshold of *average+5*SD* (see §1.6.6.2 for details) applied over the number of articles published by each author over the four years around the editor’s appointment (i.e. the two years before the editor’s appointment and the two years after).

We then ran a linear mixed model. The difference in the number of articles published in the editor’s journal between before and after the editor’s appointment was modeled as a linear function of:

1. The type of authors (i.e. editor’s co-authors /other authors)
2. The period considered during which the articles were published (i.e. before/after the setup of the non-financial conflict of interest policy)
3. The number of articles published by the author during the two years before the editor’s appointment,
4. The two-way interaction between regressors 1 and 2.

Editors were set as a random effect over the intercept. Regressor 4 captures a difference in the publications of editor’s former co-authors after the setup of the non financial conflict of interest policy. A statistically significant result for regressor 4 can be interpreted as the presence of non-financial conflicts of interests between editors and their former co-authors.

We checked whether the exclusion of editors appointed before September 16^th^ 2011 in this analysis biased the results. We reran the same analysis by including also editors who were appointed before September 16^th^ 2011. We performed the same robustness analyses as in §1.9.3.1.

#### Comparison of changes in the number of publications by authors with the same research affiliation as the editor’s previous ones in the editor’s journal and the other Nature journals before and after the setup of the non-financial conflicts of interest

This analysis complements the one of §1.9.3.6. We used the same inclusion procedure for editors as in §1.9.3.6: we excluded from the analysis those editors who were appointed between May 2015 and May 2017 and before September 16^th^ 2011. 71 editors were thus included in this analysis. These editors are a subset of the 111 editors of the analysis of §1.9.3.3. 91 editors were included in the analysis when considering editors who were appointed before September 16^th^ 2011 in the robustness analyses (see §1.11 and §2.3).

Using the total number of articles with at least one author affiliated to one of the editor’s former research institution, we ran a two-sample t-test between the difference between the two left bars of figure 3C and the difference between the two right bars. Differences were computed by pairing data per editor.

We checked whether the exclusion of editors appointed before September 16^th^ 2011 in this analysis biased the results. We reran the same analysis by including also editors who were appointed before September 16^th^ 2011. We performed the same robustness analyses as in §1.9.3.3.

### Limits of our analyses

We did not include the full dataset of submissions to *Nature* journals because these data are not publicly available. One may argue that these data may change completely our results. This is very unlikely. First, this explanation would account only for part of our results: it could hardly explain the results with new authors and the impact factor (figures 2B, 2C and 3A). An explanation at the level of editors instead of authors accounts for all our results. Second, the analysis of submission data introduces other biases. Submissions have more likely a higher heterogeneity in terms of quality and outstandingness than publications. Quality and outstandingness are major criteria of selection in *Nature* journals [12]. They are also difficult to control in the analyses. Therefore, any difference in results between submissions and publications may be related to a difference in quality or outstandingness between the two dataset without contradicting our results. Third, the only dataset of submissions at *Nature* journals that we could analyze confirmed rather than contradicted that our results are associated to editors. Fourth, the hypothesis of a change of submission behavior of co-authors to explain the increase in publications of editor’s co-authors in the editor’s journal was not supported by those editors whose co-authors only published in the editor’s journal before the editor’s appointment (see §1.10.7 and §2.4.5).

Since there is no current rule for reporting authors’ affiliation in articles, the quality of these data might have been highly heterogeneous between articles: the same author might have reported his/her affiliation differently in two articles (e.g. adding the zip code of the institution in one of them) or the affiliation of all authors of a given article might have been reported only for one of the authors because all authors of the article shared the same affiliation. Although this heterogeneity was addressed with the use of keywords for the affiliations of editors in our computations, we might have missed authors with the editors’ affiliation because of missing data. Additionally, this heterogeneity did not allow to compare between institutions the number of articles published by their researchers especially for research institutions where the editors worked as a researcher before joining *Nature* journals.

We did not take into account co-first or co-last authors in our analyses. Taking this information into account would not add much to our results: two co-first authors must still decide which one of the two will appear first in the order of authors on the article even though they add an asterisk to mention that they are co-authors. Additionally, taking into account this information in the analyses would have substantially increased the number of analyses: we would have had to compare the results of the analyses without taking into account co-first and co-last authors (i.e. these analyses are the ones reported in the current article) to the results of the analyses taking them into account. Considering the robustness of our results, we may speculate that these additional analyses would have given results consistent with those reported here.

We could not match articles with the editors who handled them. One could object that our results cannot therefore be associated with editors. However, editors of *Nature* journals meet regularly to discuss the submitted manuscripts. They are also requested to liaise with editors of the other *Nature* journals (see §2.4.3). These exchanges between editors allow the influence of the other editors over the handling editor of a given manuscript. Such influence is not captured by a match of articles with their handling editor. Additionally, we showed associations between several of our results and editors’ characteristics. Besides these associations, our results were also tied to editors: we analyzed the publications of editors’ co-authors and authors affiliated to one of the editors’ former research institutions, we focused on time periods around the editors’ appointment at *Nature* journals and, in other analyses, we tailored the period of publication to the editor’s period of work at *Nature* journals. Therefore, matching publications with their handling editor has little chance to modify or add much to our results.

The selection of editors may have biased the results. For instance, there may have been editors appointed before the setup of the non-financial COI policy who left *Nature* journals by December 2020 (date of data collection) while editors appointed after the setup of this policy may be more numerous (see §2.2.4.2). We therefore quantified in §1.10.9 and §1.10.10 potential biases due to the selection of editors. Although these analyses cannot rule out such selection biases, they suggest that biases of our results due to the selection of editors are limited.

The name of authors might have contained special characters which might not be referenced in the ASCII code. Additionally, the name of an author containing these special characters might have been spelled differently in two articles (e.g. the special character is used in one article and the closest regular character is used in the other article). Considering the number of special characters taken into consideration in our computations (72 special characters impacting letters a, c, e, i, n, o, s, u and y either lower case or upper case) to address this issue, cases of authors with special characters who would not have been addressed might have been limited and might not have biased much the results.

The name of editors may be spelled differently in the *Nature* and LinkedIn websites. In that case, we kept the name of editors mentioned in the *Nature* website because: *Nature* journals deal with academic researchers while LinkedIn not and it is important for researcher for their career to have their publication associated to their name without errors (the ORCID identifier has been built for that purpose).

The family name of female, and sometime male, editors may have changed across time. It also applies to editors’ former co-authors and authors in general. This issue is part of the author name disambiguation problem [19]. While we addressed the issue of homonyms, we could not quantify the effect of changes of author’s names over our results. In our case, we performed an adaptive filtering for the homonyms. This filtering was based on the number of articles published by each author. Therefore, authors who would have changed names would have, after matching their former family name with their current one, a higher or equal number of articles published than the one we used in our analyses. These authors may thus have been removed by the filtering addressing homonym issues before the statistical analyses. We may thus speculate that our results would not change much if we had controlled for changes in family names.

Several analyses (see §1.9.3.1, §1.9.3.2 and §1.9.3.6) included only part of the 190 editors considered for statistical analyses. Although the criteria used to include editors in these analyses were independent from the goal of the analysis, they might have introduced biases in our results. Likewise missing data for editors may have created recruitment biases. However, these biases are likely extremely limited. The 190 and the 306 editors were very similar for almost all editorial characteristics (§2.1.3). Subsamples of editors had similar characteristics (see §1.11 and §2.3) or had differences which explained why they were included in the analysis but without showing a bias in the sampling that would have biased the results (see §1.11 and §2.3). The number of editors included in each analysis was substantial and the number of editors for whom no starting date at *Nature* journals could be retrieved was limited to 4.98% of the sample.

We did not consider the duration of the publication process in the definition of new authors. Therefore an author who published two articles in *Nature* journals with an interval of a couple of weeks between the two publication dates was considered as a known author for the second article in our analyses while the editor making the decision of acceptance of the second article might have not been aware of the first article. This case may have biased our results. However, the bias is likely extremely limited. This case would have created a bias only if the two articles were not processed by the same editor. Because editors are mainly specialized in a specific research field and most *Nature* journals included in the study were specialized, the two manuscripts submitted by the same author in a *Nature* journal would have had high probability to have been processed by the same editor. Moreover, our results relied on slopes (figures 2B and 2C) and slopes were computed over the entire time range of publication of the journal (figure 2C) or the entire time range of work as editor for a given editor except the first year (figure 2B). Therefore, the bias due to the case of two articles submitted a few weeks apart by the same author would have likely introduced noise in the fits of the slopes rather than biased the slopes, reducing thus only the statistical power. Additionally, the robustness analyses showed that the slopes were overall similar among all durations of the time window used to define new authors (data not shown but available at https://entrepot.recherche.data.gouv.fr: <https://doi.org/10.57745/3QL466>

).

Identifying editors’ former co-authors allows to capture editors’ ties with authors. However, this method does not account for all ties the editor may have built during his/her research career. It does not also account for the quality of the relationship between the editor and these authors. Editors and their former co-authors may not always have good relationships even though they published together. Additionally, editors may have become friends with other researchers during their academic career but without publishing with them. These friends may not work in the same research laboratory but in a research field which is close to the one of the editor. This latter tie with the editor is not captured with our method. These researchers may have published articles in *Nature* journals after the editor’s appointment at *Nature* journals. Articles of these authors have thus been grouped in our analyses with the articles of authors who were not editors’ former co-authors. Considering our results of figure 2A, we therefore can speculate that addressing this wrong classification of authors would increase, rather than decrease, the difference reported in figure 2A between authors with ties to the editors and other authors.

Our algorithm could not extract any article published in *Nature Ecology & Evolution* referenced in PubMed although a direct search in the PubMed website retrieved articles from this journal when filling the field “journal” with the journal name “*Nature Ecology & Evolution*”. We did not face this problem with any other *Nature* journal included in the study. This failure comes likely from the fact that our algorithm considered that an article was published by one of the *Nature* journals included in the study if the journal’s name referenced in PubMed for that *Nature* journal matched exactly (case insensitive) the spelling of the name of the journal mentioned in table 1. This strict filtering aimed at preventing the inclusion of articles from journals which were not the ones included in our study but which had names containing the words of one of the *Nature* journals included in the study (e.g. articles of *Nature Review Neuroscience* must not be included in our analyses while articles of *Nature Neuroscience* must be). The failure of our algorithm to extract articles of *Nature Ecology & Evolution* shows that *Nature Ecology & Evolution* is not spelled in PubMed as “*Nature Ecology & Evolution*”. The problem likely comes from the character “&”. Therefore *Nature Ecology & Evolution* was not included in any of the statistical analyses addressing questions related to its publications. However, data related to *Nature Ecology & Evolution* were included in all other analyses (e.g. job offers, characteristics of editors…). Considering the results related to articles published in *Nature* journals and the number of *Nature* journals included in these analyses, missing the contribution of *Nature Ecology & Evolution* likely reduced the statistical power rather than biased the results.

Finally, authors’ names referenced in PubMed might contain errors (e.g. first and last names are reversed). This kind of error led our algorithms to consider the same author who has done this mistake in one article but not in the second one as two different authors. We could not quantify the number of these errors but we speculate that they are very limited. Authors require a visibility in the databases to be known. Such errors penalize them.

## Additional statistical analyses and models

### Characteristics of editors of Nature journals

For each editor, we computed the duration of the research experience after the doctorate by subtracting the last year of work as a researcher in a research institution and the year of the end of the doctorate. Numbers of original articles, reviews and meta-analyses as first or last authors were computed directly from the output of the second step of §1.6.3. The percentage of editors without any research experience after the doctorate in each journal was computed from the raw editors’ data (see §1.4.2).

### Analysis of the number of articles published by new authors at the launch of a new Nature journal

Results are reported in §2.2.3.

We compared the number of articles published in *Nature* the year before the launch of a new *Nature* journal to the number of articles published by new authors the year of the launch in *Nature* and the new *Nature* journal. We hypothesized that the launch of a *Nature* journal would increase the space available in *Nature* and the new *Nature* journal for the publication of original articles by only new authors. We hypothesized that this increase would be seen the year of the launch of the new *Nature* journal as compared to the year before.

The rationale for this comparison relied in the generalist characteristic of *Nature*. The launch of a specialized *Nature* journal would have absorbed several articles usually published in *Nature* before the launch of the specialized journal This would thus leave room to other original articles in *Nature*. Additionally, the absorbed articles may not have filled all the space available in the new journal leaving thus an additional room for other original articles.

For each *Nature* journal included in the study except *Nature* and *Nature Communications* and using the output of the third step of §1.6.2, we computed the number of original articles published by only new authors the year of its launch. We did the same for *Nature* for the year of the launch of the new journal and summed these two numbers (i.e. number of articles in *Nature* and the new *Nature* journal the year of its launch) and called this sum N_1_. Similarly, we computed the number N_2_ of original articles published by only new authors in *Nature* the year before the launch of each *Nature* journal. This led to as many N_2_ as we had *Nature* journals besides *Nature* and *Nature Communications*. We then computed for each *Nature* journal N_1_-N_2_.

We ran a GLM in which we modeled the aforementioned difference in the number of original articles published by only new authors as a linear function of the year of the launch of the new journal. The rationale for including a time effect in the analyses rather than running a direct t-test comparison relied on the non-stationarity across years of the acceptance of articles with new first and last authors [16]. The rationale for using a GLM rather than a correlation relied in the information provided by the intercept of the GLM which cannot be captured by a correlation. This information allowed to assess to which extent, in 2020, the difference in the number of original articles published by only new authors between the year of the launch of a new *Nature* journal and the year before was different from zero.

### Association between the journal impact factor at year n-2 and the rate of articles published by new first and last authors in generalist and specialized Nature journals

General considerations: We included in this analysis only *Nature* journals which had at least three years of publication (n=25 journals) to be consistent with the analyses reported in §1.9.3.5. The goal of this analysis was to assess whether there were differences between generalist and specialized *Nature* journals. Differences conditioned which journals were included in the analysis of §1.9.3.5. Results of this analysis are reported in §2.4.2.

Using the output of the third step of §1.6.2, we computed, for each year and each *Nature* journal, the number of original articles published by new first and last authors (i.e. the number of articles by only new authors + the number of articles by known intermediate authors (i.e. with first and last authors new)). For each year and each *Nature* journal, we divided this number by the total number of original articles published over the year. We removed from the analyses the rates equal to 0 (n=2, 0.8% of the data) or equal to 100 (n=12, 5% of the data). We then ran a linear mixed model in which the IF at year n was modeled as a linear function of the rate of original articles at year n-2 by new first and last authors, the type of journal (i.e. generalist/specialized) and the two-way interaction. The journals were set as a random factor over the intercept. The rationale for shifting by two years the IF and the number of articles published was that the IF at a given year is computed with the citations of the articles published over the previous two years.

### Analysis of retracted articles

Results are reported in §2.2.5.

Result 1: the percentage of retracted articles in *Nature* journals remained stable across years.

Using the output of §1.6.4 and the output of the second step of §1.6.2, we computed, for each *Nature* journal separately and for each year starting from 1990 until 2020 the ratio of the number of initial articles which have been retracted to the number of original articles published that year. We shortened the period from 1990 to 2020 by the average duration of retraction over the journal rounded to the upper year. Removing the average duration of retraction within each journal allowed to remove from the analyses the years during which future retracted articles of that journal were still not retracted. Including such years would have biased our results: it increases the chances to have a decreasing rate of retracted articles over years. If no retraction duration could be computed for a given *Nature* journal, this latter was set to zero.

We then ran a linear mixed model in which the yearly rate of retraction was modeled as a linear function of time (i.e. years elapsed since 1990), the journal type (i.e. generalist/specialized) and their interaction. The journal was set as a random factor over the intercept.

Result 2: among retracted articles, there were more retracted articles by authors who had already an article retracted than retracted articles among original articles.

We used the retraction date (i.e. the date of the retraction note) as a lower bound for the date of publication of a second retracted article. This is summed up below:

Lower bound

Duration of retraction

Time

Second article retracted (date of publication of the retraction note)

Second article published by at least an author of the first article

First article published

First article retracted (date of publication of the retraction note)

The rationale for this lower bound was that the publication of a retraction note guaranteed that, at the time of the editorial decision of acceptance of the second article, all editors had the opportunity to be aware of the retraction of the first article.

For each retracted article (this article is referred to retracted article 1 thereafter), we checked whether its authors did not publish any retracted article after the lower bound of retracted article 1. If no author of retracted article 1 contributed to any future retracted article, the number of retracted articles for which none of their authors had another retracted article later was incremented by one. In the other case (i.e. at least one author of retracted article 1 was also author of a retracted article published after the lower bound of retracted article 1), the number of articles by authors who had already contributed to a former retracted article was incremented by one. Note that this increment is made irrespectively of the journal. Additionally, for each year and each journal in which there was at least one retracted article, we computed the total number of original articles published that year and summed all these numbers over all journals. We finally computed the ratios:

$$\frac{N retracted articles by authors who had already contributed to a former retracted article}{N retracted articles by authors who had never contributed to another retracted article}$$

And:

$$\frac{N retracted articles by authors who had never contributed to another retracted article}{N original articles over the same years}$$

We compared these ratios with a Fisher exact test. Note that retracted articles 1 included only articles for which a duration of retraction could be computed. Additionally, there was no case of an author of a second retracted article (this article is referred as retracted article 2 thereafter) after retracted article 1 who would have been also author of a third article published after the lower bound of retracted article 2 and which would have been retracted thereafter (likewise retracted article 1 and retracted article 2).

### Analysis of job offers for editorial positions at Nature journals

We mainly characterized the data of the job offers by computing percentages. Results are reported in §2.2.4.

We also tested whether job offers which stated that candidates with a “strong research record” and a “postdoc were preferred” were associated with the research and editorial experiences of the editorial board of the journal where the candidate would be appointed. The rationale is that the editor in chief may likely write the job offers and ask for feedbacks from his/her board. Note that a strong research record is a subjective assessment. It may be independent of having a research experience after the doctorate. Thus candidates who do not have any academic research experience after their doctorate but who have published several articles during their doctorate may be considered as having a strong research record.

Using the output of the second step of §1.6.3 and for each editor included in the analyses, we computed his/her number of years as editor at *Nature* journals (i.e. an editor of a given *Nature* journal may have been editor in another *Nature* journal before). Then, for each journal, the mean was computed over the editorial board of the journal. We did the same for the time spent in academic research and the number of editor’s original articles published as first or last author. We thus obtained for each *Nature* journal:

- the average duration of the editorial experience of its editorial board,
- the average duration of the scientific experience of its editorial board,
- the average number of original articles published as first or last author by the journal editorial board during the editors’ academic research career.

We ran a logistic regression to test whether the editorial board’s research experience, editorial experience and number of original articles published as first or last author by the editors were associated with the statement, in the job offers for editorial positions, that a postdoc was preferred or that a strong research record was preferred. The category of job offer (job offers with explicit statement that a postdoc position or a strong research record was preferred/other job offers) was modeled as a logit link of the average time spent in academic research (in years) of the editorial board of the journal issuing the job offer, the average duration spent as editor at *Nature* journals by the editorial board of the journal issuing the job offer (in years) and the average number of original articles published as first author by the editorial board of the journal issuing the job offer. This model was a mirror of the analysis of §1.9.3.4.

### Association between journals impact factor and the prestige of Nature journals

*Nature* journals editors divided *Nature* journals into three categories of prestige [25]. *Nature* was set in the first category with the highest prestige. Specialized *Nature* journals were set in the second category with intermediate prestige. *Nature Communications* was set in the third category with the lowest prestige. This analysis aimed at testing whether the impact factor (IF) discriminated these three categories.

We ran two linear mixed models. The first one compared the IF of *Nature* to the specialized *Nature* journals. Because IF changes over time, we analyzed the entire history of IF. For each specialized *Nature* journal and for each year of release of its IF, we computed the difference between the IF of *Nature* that year and the IF of the specialized *Nature* journal. We then model this difference as a linear function of time. Journals were set as a random factor over the intercept. The time regressor was computed as follow:

- 0 for 2019
- -1 for 2018,
- -2 for 2017,
- -3 for 2016…

The intercept of the model captured thus the average difference in IF between *Nature* and specialized *Nature* journals in 2019. We hypothesized that this difference was positive: *Nature* is considered having a higher prestige than specialized *Nature* journals [25].

The second model was the same as the first one except that the difference in IF was computed between specialized *Nature* journals and *Nature Communications*. We hypothesized that this difference was positive: specialized *Nature* journals are considered having a higher prestige than *Nature Communications* [25].

### Analysis of the consequence of a change in submission behavior of authors over their publications in the editor’s journal

This analysis aimed at testing whether a change in submission behavior of authors may have contributed to the increase in publications of editor’s former co-authors. The rationale for such a change in behavior would be that former co-authors of the editor may submit more articles after the editor’s appointment with the hope that they may publish more in the editor’s journal. Such a difference in behavior would be typically observed among co-authors who did not publish in the editor’s journal before the editor’s appointment: these co-authors would elicit publications in the editor’s journal after the editor’s appointment while they have not published before.

Therefore, among all co-authors of the editor, one would expect that the number of co-authors who published in the editor’s journal only after the editor’s appointment would be higher than the number of co-authors who published in the editor’s journal only before the editor’s appointment. This hypothesis relies on the assumption that the probability of succeeding in publishing does not change after the editor’s appointment. Within this hypothesis, the difference in publication of former editor’s co-authors after the editor’s appointment as compared to before would only be the consequence of a higher number of submissions leading to a higher expected number of publications. Of note is that a change in this probability of succeeding after the editor’s appointment would imply a role of the editor.

For each editor included in the analysis of §1.9.3.1, we computed the number of co-authors who published in the editor’s journal only before the editor’s appointment (i.e. during the same two years before the editor’s appointment as in §1.9.3.1). We did the same for co-authors who published only after the editor’s appointment (i.e. during the same two years after the editor’s appointment as in §1.9.3.1). We then divided each of these two numbers by the total number of co-authors of the editor leading to two percentages for each editor. The total number of co-authors of the editor includes co-authors who never published in the editor’s journal nor in any *Nature* journal. We compared the difference between these two percentages across editors with a paired t-test. A difference between these two percentages would support a change in the submission behavior of editor’s co-authors. We tested the robustness of this analysis by restricting the editor’s co-authors to the first group of co-authors identified with the algorithm of §1.6.6.1.

We did the same reasoning at the editor’s level. We compared editors whose co-authors published exclusively after the editor’s appointment to editors whose co-authors published exclusively before the editor’s appointment. This type of comparison differs from the previous one: editors could have co-authors who published only before the editor’s appointment but also other co-authors who published only after while here, an editor whose co-authors published only after the editor’s appointment had no co-author who published before the editor’s appointment. This analysis complements the previous one. It allows checking whether there would be any threshold effect in the results. We therefore computed the total number of editors who had at least one co-author who published in the editor’s journal during the two years before or the two years after the editor’s appointment. These two periods are the same as the ones used in §1.9.3.1. Among these editors, we computed the number of editors who had no co-author who published in the editor’s journal before his/her appointment at the journal but who had at least one co-author who published in the editor’s journal after the editor’s appointment. These editors are referred thereafter as editors of the first category. We did the same for editors who had at least one co-author who published in the editor’s journal before the editor’s appointment but who had no co-author who published in this journal after the editor’s appointment. These editors are referred thereafter as editors of the second category. We then ran a Fisher exact test to test whether the proportion (among editors with a co-author who published at least an article in the editor’s journal before or after the editor’s appointment) of editors of the first category differed from the proportion of the second category.

These two analyses (i.e. the one focused on co-authors and the one focused on editors) use the number of articles published rather than the number of articles submitted to test a hypothesis related to the submission of authors. Using publications rather than submissions allows to better control for the quality of articles. A change in the submissions after the editor’s appointment as compared to before does not guarantee that the overall quality of all articles submitted after the editor’s appointment is similar to the one of all articles submitted before the editor’s appointment.

Results of this analysis are reported in §2.4.5.

### Comparison of the publication record in the editor’s journal of editors’ co-authors and the other authors before the editor’s appointment

This analysis had two goals. The first one was to check whether editor’s former co-authors and the other authors of articles published in the editor’s journal had a similar publication record during the two years before the editor’s appointment. It justified adjusting the results of the first model of §1.9.3.1 over the number of publications before the editor’s appointment. The second goal was to complement results related to the recruitment of editors (see §1.10.5). A difference in the number of publications of editor’s former co-authors in the editor’s journal as compared to the other authors suggests that the recruitment of editors favors candidates from research groups who publish most frequently in the journal.

We used the number of articles published in the editor’s journal during the two-year period before the editor’s appointment. This number has been computed for each author for the analysis of §1.9.3.1. We discriminated the editor’s former co-authors from the other authors. Editors included in this analysis were the ones of §1.9.3.1. We addressed homonyms among authors in the same way as in §1.9.3.1 so that the data used in the current model are the same as the ones used in §1.9.3.1 for the number of publications before the editor’s appointment.

We ran a linear mixed model. The number of articles published before the editor’s appointment was modeled as a linear function of the type of author (editor’s former co-author/other author) and editors as a random effect over the intercept.

### Analysis of the association between the recruitment date of the editor and the increase in publications of editor’s co-authors and the other authors in the editor’s journal

Results of the analysis of §1.9.3.1 may have been biased by the election of editors (see §1.1.2). Editors appointed in the past who were referenced on the *Nature* website when we collected the data (see §1.1.2) were those editors who have not left *Nature* journals in the meantime. On the opposite, none of the editors recently appointed had likely left the journal before the data collection date. Editors who left the journal before the data collection date could not be included in the study. The missing data of editors who had been appointed by *Nature* journals a long time ago and who left before the data collection date may have biased our results. Therefore we tested whether the increase in publications of co-authors of the editor after the editor’s appointment was associated with the date of appointment of the editor. The recruitment date of the editor was captured by the duration between the editor’s appointment and the data collection date. Note that this analysis allows disentangling the interpretation of this duration as the editorial experience (interpretation of §1.9.3.4) from the interpretation of a bias in the selection of editors. Indeed, contrary to the analysis of figure 2B (see §1.9.3.4) which investigates all the publications after the editor’s appointment until the end of 2020, the current analysis focuses only on what happens before the editor’s recruitment and shortly after (i.e. the two years after the editor’s appointment which limits strongly an effect of editorial experience over the publication outcome as compared to the analysis of figure 2B).

We thus used the data of the second linear model of §1.9.3.1 (i.e. the one with the regressors of the time spent in academic research by the editor and the number of publications as first or last author of the editor before leaving academia). We modeled the difference in the number of articles published by each author between the two years after the editor’s appointment and the two years before as a linear function of:

1. The type of authors (i.e. editor’s co-authors/other authors),
2. The time elapsed since the editor’s appointment,
3. The interaction between the two regressors.

Editors were set as a random effect over the intercept. A lack of association between the increase in publication of editor’s co-authors and the time elapsed since the editor’s appointment and the same with the other authors instead of the editor’s co-authors may be interpreted as a limited bias in the selection of editors over our results reported in the main text. These results are complemented by the results of §1.10.10.

Results of this analysis are reported in §2.4.6. We ran the same robustness analysis regarding the method used to address homonyms and the threshold values as in §1.9.3.1.

### Analysis of the association between the recruitment date of the editor and the number of articles published by editor’s co-authors and the other authors before the editor’s appointment

As in §1.10.9, this analysis aimed at quantifying a potential bias due to the selection of editors. Contrary to the analysis of §1.10.9, we focused exclusively on publications before the editor’s appointment. Therefore, the time elapsed between the editor’s appointment and the date of data collection cannot be interpreted in this analysis as editor’s editorial experience.

We used the data of the linear model of §1.10.8. We modeled the number of articles published by each author before the editor’s appointment as a linear function of:

1. The type of authors (i.e. editor’s co-authors /other authors),
2. The time elapsed since the editor’s appointment,
3. The interaction between the two regressors.

Editors were set as a random effect over the intercept. A lack of association between the number of publications of editor’s co-authors and the time elapsed since the editor’s appointment may be interpreted as a limited bias in the selection of editors over our results reported in the main text. These results strengthen the ones of §1.10.9. It also suggests that the association reported in figure 2B may be interpreted as an association with the editor’s editorial experience and not with a bias due to the selection of editors.

Results of this analysis are reported in §2.4.7. We ran the same robustness analysis regarding the method used to address homonyms and the threshold values as in §1.10.8.

## Control and robustness analyses

These analyses were additional controls to the ones that were set in the statistical analyses reported in supp. §1.9 and §1.10.

### Control analyses

These analyses checked that:

- the identification of original articles among publications in *Nature* were was more homogenous over the period considered for the analyses (i.e. January 1^st^ 1990 to December 12 2020) than before January 1^st^ 1990,

- the ranking in the authorship for articles published in Nature was different from an alphabetical ordering in order to justify the categorization of authors as first, last or intermediate according to their position as author in the article,

- the sampling of job offers has missed a limited number of job offers that has likely not biased the results.

The methods and results are not shown but they are available at https://entrepot.recherche.data.gouv.fr: <https://doi.org/10.57745/3QL466>.

### Robustness analyses

These analyses aimed at checking that the criteria used to select editors, to address homonyms, to compute editor’s characteristics, to define new authors, and to take into account the duration of the publication process of *Nature* journals did not biased the results. For instance, for the duration of the time window used to define a new author, we checked whether results of figures 2B and 2C were also statistically significant with other lengths of the time window.

We thus ran all robustness analyses following three main axes:

1. The analyses reported in the main text were re-run but using different lengths of the time window used to define a new author: 1, 3, 4, 5, 10 years and the entire duration of publication of the journal,
2. The analyses were re-run by considering that authors whose name appeared in more than 50 articles in a given *Nature* journal could be known authors on a given article,
3. The analyses were re-run by considering different threshold in the algorithm of §1.6.6.2 used to remove homonyms among authors: *Average+3*SD*, *Average+4*SD*, *Average+6*SD*, *Average+7*SD*.

The second axis used one or the two following considerations, whenever appropriate:

- Different durations for the publication process: 6, 7, 9 and 10 months. For each of these durations, we re-ran the analyses reported in the main text in which the duration of the publication process was considered. We did not perform this robustness analysis for the analyses of §1.9.3.1 and §1.9.3.6 because of excessive computational time (around 6 months for each editor).
- The use of a more stringent algorithm to identify homonyms among editors’ names. This impacted the number of articles published as first or last author by editors before they joined *Nature* journals. It also reduced the number of editor’s co-authors. We re-ran the analyses in which editors’ co-authors or the number of articles published as first or last author by editors were used.

Additionally, the removal of editors who had homonyms or did not match the criteria of specific analyses (see §1.9.3.1 and §1.9.3.6) may have contributed to select a biased set of editors. We therefore checked that the characteristics of the excluded editors were similar to the ones of editors included in the analyses.

The methods and results are not shown but they are available at https://entrepot.recherche.data.gouv.fr: <https://doi.org/10.57745/3QL466>.

## Specific considerations for the identification of editors without any research experience after the doctorate, the identification of conflicts of interests and the analysis of retracted articles

For all editors for whom a research experience after the doctorate could not be identified from the *Nature* website (see §1.4.2), we checked that the editor had no affiliation to a research institution between the end of his/her doctorate and his/her recruitment as editor at *Nature* journals. We used the editors’ Linkedin and ORCID profiles and Google searches with the editor’s name complemented by the words “*Nature* editor”.

The programs for the analyses of the publications of editors’ co-authors and authors affiliated to an editors’ former research affiliation (see §1.9.3.1) were manually checked a second time while all other programs were checked once (see §1.6.9).

All retracted articles for which at least one author had already contributed to a former retracted article (see result 2 in §1.10.4) were manually checked for any misclassification (e.g. the retracted article might have been directly related to the first article retracted, the retracted article might have been published before the retraction note of the first retracted article).

# Supplementary results

## Additional results of analyses reported in the main text

### First goal of the analysis reported in §1.9.3.1

One of the goals of this analysis was to quantify the contribution of the authors’ track record in the editor’s journal to the chances to publish in this journal. We tested whether this contribution differed for editor’s co-authors after the editor’s appointment as compared to before. We reported in the main text that, for each additional article published in the editor’s journal before the editor’s appointment, authors had 161% more chances to publish in the editor’s journal after the editor’s appointment. There was no difference in this odds ratio between editor’s former co-authors and the other authors (odds ratio [95% confidence interval]: 0.52 [0.24 – 1.15], p=0.11). This result is consistent with literature showing that authors who have already published as first author in a journal have higher chances to publish subsequently as last author in that journal as compared to authors who did not publish as first author before in the journal [16].

### Second goal of the analysis reported in §1.9.3.1

The second goal of this analysis was to quantify whether the increase in the number of articles published by editors’ co-authors in the editor’s journal as compared to the other authors was associated with editors’ characteristics (i.e. research experience and number of articles published by the editor during his/her research career).

The increase in publications of editor’s co-authors in the editor’s journal after the editor’s appointment (results of figure 2A) was associated with the editor’s time spent in academic research (beta value (SEM): 0.11 (0.026), p=0.00003). However it was not the case with the increase in publications of the other authors (beta value (SEM): 0.012 (0.011), p=0.25). This difference was statistically significant (p=0.00005). The number of original articles published as first or last author by the editor during his/her research career was not associated with any of the two aforementioned increases in publications in the editor’s journal (beta value (SEM): -0.02 (0.042), p=0.64; 0.006 (0.015), p=0.69 respectively). These results are consistent with the ones of figure 2B: the results of the analyses of publications in *Nature* journals (i.e. the increase in publications of editor’s co-authors in figure 2A and the decrease in publications of new authors in figure 2C) are associated with the time spent by the editors in academic research but not with the number of original articles published by the editors as first or last author during their research career. That the signs of the associations are opposite (i.e. the increase in publications of editor’s former co-authors is higher for editors with more experience in research while the decrease over time in the rate of articles with only new authors is faster for editors with a lower experience in research) does not make the two results inconsistent. It may signal two different types of shortcomings.

### Analysis reported in §1.9.3.3

This analysis aimed at quantifying the variations in the total number of articles published by authors affiliated to one of the former research institutions of the editor after the editor’s appointment as compared to before. We compared these variations between the editor’s journal and the other *Nature* journals.

Results are reported in supplementary figure 2A.

### Analysis reported in §1.9.3.4

Results reported in figure 2C were consistent with previously reported results [16, 26].

### Analysis reported in §1.9.3.5

General consideration: this analysis was restricted to *Nature* and *Nature Communications*. The rationale is that these two journals elicited the strongest association between the rate of articles published by new first and last authors at year n-2 and IF at year n (see §2.4.2). These two journals had therefore the highest incentive to act on the acceptance rate of articles by new first and last authors.

Additional result 1: the difference between new first and last authors and the other authors reported in the two left bars of figure 3A has been increasing since 1997.

We ran the same linear mixed model as the one used in result 1 (see result 1 of §1.9.3.5) except that we added an interaction with the difference in time between 2020 and the year of publication considered. This difference captured a time effect and locked the intercept of the model in 2020. We considered a duration of two years to characterize new authors (see §1.5.1).

The modulation of the variations in the number of articles of new first and last authors by the variation of the IF increased across years (beta (SEM): 2.24 (0.81), p=0.006, supplementary figure 2F (the red bar on the left)). However, it was not the case for the articles with at least a known first or last author (beta (SEM): 0.28 (0.81), p=0.34, supplementary figure 2F (the blue bar on the left)).

Additional result 2: there was no association between IF variations and the total number of submitted articles.

The number of original articles submitted to *Nature* was not impacted by variations of the IF (slope (SEM): -0.88% per point of IF (0.94), p=0.36; this percentage represents the ratio of the difference between year n+1 and year n in the number of articles submitted to the number of articles submitted at year n). However, fewer published articles were referenced in the computation of the IF the year after a decrease of the IF as compared to the year before (slope (SEM): 2.04% per point of IF (0.94), p=0.04; this percentage represents the ratio of the difference between year n+1 and year n in the number of articles submitted to the number of articles submitted at year n). This result was consistent with previous reports [27-29]. The difference between submissions and published articles (p=0.04) suggests a role of the editorial process in the associations between IF variations and the distribution of published articles rather than a change in the submission behavior of authors.

### Analysis reported in §1.9.3.7

The absence of difference in figure 3C between before and after the setup of the non-financial COI policy is consistent with the editorial policy which excludes from the review process those reviewers affiliated with the same institution as one of the authors of the manuscript [12]. This policy applies to the date of submission of the manuscript. However, this policy does not exclude that these reviewers had been working in the same institution as the authors in the past. Thus this situation is the same for *Nature* journals editors: they are not affiliated with their former research institutions at the time of the publication of the manuscript of authors affiliated with these institutions.

## Results of additional analyses

Analyses of this paragraph are reported in §1.10.

### Nature journals have only professional editors.

86% of the analyzed job offers were explicitly full-time positions. Only one journal (4% of journals which issued job offers for editorial positions aiming at making decisions about the submitted manuscripts) issued all its job offers (n=2 job offers) without the statement that the position was full-time. There was no job offer which mentioned a part time position nor a weekly duration of work of less than 35 hours. These results suggest that all editorial positions at *Nature* journals are full-time consistently with *Nature*’s claim [13]. Job offers without these statements are therefore likely the consequence of a missing information.

### IF is associated with the prestige of the journal

*Nature* editors have claimed that *Nature* journals are grouped into three categories of prestige. *Nature* is in the highest [25]. Specialized *Nature* journals are in the middle category [25]. *Nature Communications* is in the lowest category [25]. The prestige of these journals is also claimed by *Springer Nature* in the job offers for editorial positions at the *Nature* journals included in the study (supp. §2.4.1).

The statistical analysis associated to the following results is reported in §1.10.6.

Since 1997, the IF of *Nature* has been above the average IF of the specialized *Nature* journals included in the study. This difference has been increasing over the years (slope (SEM): 0.37 (0.06), p<0.00001; intercept in 2020 (SEM): 19.1 (1.95), p<0.00001; supplementary figure 2B). The intercept captures the difference between *Nature* and the specialized *Nature* journals.

Since the launch of *Nature Communications* in 2010, its IF has been below the average IF of the specialized *Nature* journals included in the study. This difference has been stable across the years (slope (SEM): -0.1 (0.09), p=0.28; intercept in 2020 (SEM): 11.5 (2.08), p=0.00001; supplementary figure 2B). The intercept captures the difference between the specialized *Nature* journals and *Nature Communications*.

These results suggest that IF captures the difference in prestige between journals.

### The launch of a new Nature journal is not associated with more articles published by only new authors

The statistical model of this analysis is reported in §1.10.2.

Launching a new *Nature* journal increased the likelihood of articles by only new authors to be published: more original articles of only new authors were published in the year of the launch (in *Nature* and the new journal) as compared to the year before (in *Nature*). However, this likelihood consistently approached zero for the launch of the most recent new *Nature* journals (slope (SEM): -3.47 articles/year (1.33), p=0.01; intercept in 2020 (SEM): -23.6 articles (17.7), p=0.2; supplementary figure 2C). This result suggests that the launch of a new journal does not contribute to explain the difference in publications between new authors and known authors. Launching a new journal increases mathematically the total number of articles published by *Nature* journals. New authors may thus have more opportunities to publish the year of the launch: their publications may be in the existing *Nature* journals if not in the new journal.

### The current recruitment policy of editors at Nature journals contributes to increase rather than decrease the differences in publications between authors with different reputation to the editors

This statement is the conclusion of:

- the consistency between editors’ characteristics limiting biases in publications (figure 2B) and the content of the job offers for editorial positions at *Nature* journals,
- whether editors had established co-authors in the journal shortly before their appointment while their co-authors elicit a higher increase in publications after the editor’s appointment as compared to the other authors (figure 2A).

First, a limited number of job offers for editorial positions at *Nature* journals request explicitly a research experience after the doctorate and a strong research record. Job offers for journals with editorial boards with the lowest editorial experience and the highest academic experience lack these statements more often (supplementary figures 3A and 3B). Additionally, among editors included in the analysis reported in §1.9.7, those recruited after the setup of the non-financial COI policy had a similar research experience to those recruited before (see §1.11 and §2.3). Moreover, editors who stay at *Nature* journals have a lower scientific experience than the newly appointed editors suggesting that the most experienced editors leave faster *Nature* journals.

Second, former co-authors of the editors published more in the editor’s journal during the two years before the editor’s appointment than the other authors. It suggests that editors are recruited among the most prolific research teams in the journal.

Third, a quarter of job offers for editorial positions at *Nature* journals have to be advertised again at least once at the end of the first period of advertisement.

However, *Nature* claimed in 2022 that *Nature* journals would work to increase the diversity of their editors [30]. This change does not aim at addressing conflicts of interests nor the research experience of candidates for editorial positions. Therefore, it is unclear that this change will lead to an improvement of the results we report. Assessing the consequence of this change requires a specific study beyond the scope of our article.

#### Requested characteristics for candidates applying for job offers for editorial positions at Nature journals and association with the characteristics of the editorial board

The association with the characteristics of the editorial board is assessed with the logistic regression reported in §1.10.5.

The differences in publications between authors with distinct reputational inputs to the editors may be reversed when new editors with more scientific experience are appointed. However, 27% of job offers posted between December 2020 and December 2021 for editorial positions at *Nature* journals stated that “a postdoc is preferred” together with “a strong research record is preferred”. This happened more likely in the job offers issued by *Nature* journals with editors having the lowest editorial experience or the longest time spent in academia (relative risk [95% confidence interval] for each increase of one year of the editorial experience: 1.18 [1.02 – 1.37], p=0.03; relative risk for each increase of one year of the time spent in academia: 0.53 [0.3 –0.93], p=0.03).

Consistently with the results of figure 2B, that both “a postdoc is preferred” and “a strong research record is preferred” were stated in the job offer was not associated with the number of original articles published by the editorial board before joining *Nature* journals increased (relative risk of a lack of statement [95% confidence interval]: 2.17 [0.76 – 6.21], p=0.15).

#### Relationship between the launch of new journals and the recruitment of editors

The faster increase in the release of new journals by the *Nature* family of journals requires the recruitment of enough editors. While the analysis of job offers suggests that *Nature* journals may hire editors with a relatively limited scientific experience (table 1) in line with their recruitment policy [31], the large number of editors without any research experience after the doctorate may be related to a difficulty in finding adequate candidates. Indeed, 23% of the job offers had to be advertised at least a second time (mean number of advertisements of job offers which had to be advertised at least a second time (SD): 2.76 (0.97)) after the closing date for applications. Additionally, 24% of editors making decisions about submitted manuscripts had to be renewed each year. Thus despite this difficulty in finding candidates, the rate of editors leaving *Nature* journals, and 22% of editors of the editorial board without any research experience after the doctorate (PhD or MD), the *Nature* family of journals continues creating new journals: 2 in 2022, i.e. *Nature Cardiovascular Research* and *Nature Synthesis*, and 2 in 2023, i.e. *Nature Mental Health* and *Nature Water*, for which 8 job offers were collected and 2 of them were republished.

#### Editors who stay at Nature journals have a lower scientific experience than the newly appointed ones

Editors without any research experience after the doctorate tended to be working longer for *Nature* journals as compared to the others (mean (SD): 7.68 years (8.23) vs 5.86 years (5.76), p=0.06). Consistently, editors having published fewer original articles as first author have been working longer for *Nature* journals (r_Pearson_=-0.17, p=0.02). This result was robust when using the first group of articles identified with the algorithm of §1.6.6.1 (r_Pearson_=-0.2, p=0.005). This suggests that the most scientifically experienced editors are not the ones who stay at *Nature* journals.

#### Comparison of the publication record of editor’s former co-authors before the editor’s appointment with the record of the other authors

The model of this analysis is described in §1.10.8. Former co-authors of editors published on average more articles in the editor’s journal during the two years before the editor’s appointment than the other authors (mean (SEM): 0.8 article (0.05) vs 0.55 article (0.02), p<0.0001). This suggests that candidates coming from the most prolific research teams in the journal recruiting editors have higher chances to be hired. Additionally, this result justifies adjusting for the publication record of authors in the analysis of §1.9.3.1.

### The attention paid to the content of the articles published in Nature journals may not contribute to the differences in publications observed between authors with distinct reputational inputs to the editors

This analysis relied on retracted articles. The retraction of an article occurs when a major flaw in the scientific quality of the article has been identified after publication [29]. One may thus expect that editors may pay an extra attention to new submissions of authors who already had a retracted article in the past. The reduction over years of the rate of articles published by new authors reported in figure 2C may be the consequence of an increase in the attention paid to the content of manuscripts. The same consideration may apply to the results with the IF (see figure 3A).

For all *Nature* journals, the percentage of retractions per year has remained stable over the last 30 years (slope (SEM): -0.011%/year (0.014), p=0.45 for general *Nature* journals; -0.005%/year (0.005), p=0.34 for specialized *Nature* journals; supplementary figure 2D and [32]). This progression differs from the one of original articles published by new authors only (figure 2C). Moreover, 2.5% of retracted articles had at least one author who contributed to a former retracted article. Retraction of the first article was public at the time of publication of the second retracted article. However, only 0.18% of original articles lead to a retraction. The difference between these two classes of retraction (2.5% vs 0.18%) was significant (p=0.0002).

These results suggest thus that the attention paid by the actors of the publication process to the content of manuscripts does not contribute to the reduction over time of the rate of articles by new authors (figure 2C) nor to the difference in the evolution of publications between new authors and established authors after a decrease in IF of the journal (figure 3A).

### Editors of Nature journals are requested to liaise extensively with researchers

91% (71 out of 78) of job offers for editorial positions aiming at making decisions about submitted manuscripts to the *Nature* journals included in the study mentioned that the recruited editor would liaise extensively with the researchers worldwide, meet them to learn more about their research and make *Nature* better known to them, or visit laboratories. 84% (21 out of 25) of *Nature* journals issuing job offers for editorial positions aiming at making decisions about submitted manuscripts to the *Nature* journals issued at least one job offer with one of these statements. However, we could not find in these job offers nor on the *Nature* website which rule would underlie the choice of researchers and research institutions to be visited. The names of institutions and researchers visited by editors of *Nature* journals are not disclosed. Interactions with specific members of other companies are acknowledged as possible sources of conflicts of interests [33, 34].

## Results of the robustness analyses

Editors not included in the main analyses had scientific and editorial experiences similar to the ones of editors included in the main analysis. In parallel, all results reported in the main text were robust with regards to:

- a length of the time window used to define new authors of two to four years included,

- a time shift to take into account the duration of the editorial and publication processes of six to ten months after the article submission,

- the methods used to tackle homonyms among authors and editors,

- the thresholds used in the methods to tackle homonyms among authors and editors.

The selection of editors was not associated with a bias in the main results nor with the characteristics of editors.

Therefore, the results reported in the main text had little chance to have been biased because of the choices made in the analyses. Additionally, the substantial number of editors discarded from the analyses because of homonyms supports the systematic assessment of the robustness of results in studies where homonyms may bias results.

## Results of analyses mentioned only in the supplementary materials

### Prestige of Nature journals in job offers for editorial positions at Nature journals

Job offers for editorial positions handling original research manuscripts at *Nature* journals contain statements of different nature which relate to prestige. Thus, 69% of the analyzed job offers which were dedicated to an editorial position in *Nature* journals explicitly mentioned that editors would publish “the world’s best research articles” or “the world’s highest impact research articles” or that “the journal is the world’s leading multidisciplinary journal” or “is the world’s leader in publishing high quality scientific articles” or that “the journal publishes the most significant advances, exceptional scientific advances or articles of the highest quality and significance” or that “the journal is prestigious”. This result is consistent with the claim on the *Nature* website that *Nature* publishes the “finest peer-review research” [13].

### Relationship between the rate of original articles of new first and last authors and subsequent IF

The statistical model of this analysis is reported in §1.10.3.

In both generalist and specialized *Nature* journals, an increase in the rate of original articles of new first and last authors was associated with a decrease in the IF two years later. This relationship was stronger in generalist *Nature* journals (p=0.05, supplementary figure 2E). Consistently with literature, articles of new authors are cited less frequently than those by established authors [16]. This analysis justifies focusing on *Nature* and *Nature Communications* to investigate the effect of variations of IF over subsequent variations in the rate of original articles published by new first and last authors. These results are reported in figure 3A.

### Justification of the consideration of all articles published in all Nature journals in the definition of new and known authors and justification of not tying editors to the manuscripts they handled

We defined authors who were known to the editor of a given *Nature* journal as authors who had already published in any of the *Nature* journal included in the study. We did not restrict this definition to the journal of the editor. The rationale is the extensive liaising of editors with editors of other *Nature* journals which gives to the editors the opportunity to exchange about authors of their journal with editors of other *Nature* journals. This extensive liaising was explicitly mentioned in job offers released by *Nature* journals for editorial positions aiming at handling submitted manuscripts.

93% of the analyzed job offers which were dedicated to an editorial position in the two generalist *Nature* journals (i.e. *Nature* and *Nature Communications*) mentioned explicitly that editors will be liaising extensively with the editors of other *Nature* journals. This proportion was not statistically different from 100% (p=0.24). However, only 18% of the job offers which were dedicated to an editorial position in the specialized *Nature* journals had this explicit statement: this rate differed from the one of the generalist *Nature* journals (p<0.0001). This suggests that the communication between editors of distinct *Nature* journals goes through the two generalist *Nature* journals. These exchanges between editors of distinct *Nature* journals justified the definition of new authors (see §1.5.1) as authors who have not published in any *Nature* journals rather than in a specific *Nature* journal.

A similar reasoning may apply to the name of the editor handling a given manuscript. The high exchanges between editors within a journal or between *Nature* journals allow other sources of suggestions to the handling editor.

### Justification of the consideration of the training period in the exclusion period of publication after the editor’s appointment

52% (13 out of 25) of *Nature* journals which issued job offers for editorial positions aiming at making decisions about submitted manuscripts issued at least one job offer in which it was explicitly mentioned that a full editorial training would be provided to candidates without any editorial experience. However, only 27% (20 out of 73) of job offers for positions which accepted candidates without any editorial experience (i.e. job offers for associate or senior editors) had this explicit statement. This result justifies the consideration of the duration of the training of editors in the time shift used in the third step of §1.6.3. However, *Nature* journals did not provide in their job offers any information on the content or the duration of this training.

### Lack of association between a change in submission behavior of authors and the increase in publications of editor’s former co-authors

This analysis is reported in §1.10.7.

Only 56 editors out of the 153 considered in the analysis of §1.9.3.1 had at least one co-author who published in the editor’s journal either before or after the editor’s appointment. This proportion suggests that there may be a limited change in the submission behavior of authors after the editor’s appointment.

The 56 editors had on average 4.95% (SD=5.91%) of their co-authors who published only after the editor’s appointment and 4.44% (SD=6.12%) who published only before. These two proportions were similar (p=0.66). Similar results were obtained when using the first group of co-authors of the editor (mean (SD): 6.55% (7.87%) vs 6.86% (9.22%), p=0.72). Of note is that, on average, only 12.1% (SD=10%) of editor’s co-authors published before or after the editor’s appointment (17.95% (SD=12.85%) when restricting editor’s co-authors to the first group identified with the algorithm of §1.6.6.1).

17.9% of the 56 editors were editors whose co-authors published in the editor’s journal only after the editor’s appointment. On the other side, 12.5% were editors whose co-authors published only before the editor’s appointment. These two proportions were similar (p=0.6). Similar results were obtained when restricting editor’s co-authors to the first group of co-authors (21.7% vs 17.4% of 46 editors, p=0.79).

These results suggest that a change in submission behavior of editor’s former co-authors is unlikely to explain the increase in the number of publications of editor’s former co-authors in the editor’s journal after the editor’s appointment.

### Lack of association between the increase in publications of editor’s co-authors (respectively of the other authors) after the editor’s appointment and the time elapsed since the editor’s appointment

This analysis is reported in §1.10.9.

The time elapsed since the editor’s appointment at *Nature* journals was not associated with the increase in publications in the editor’s journal of editor’s co-authors after the editor’s appointment (beta value (SEM): 0.009 (0.028), p=0.75). Similar results were obtained with the other authors (beta value (SEM): 0.006 (0.009), p=0.5). Similar results were obtained with the thresholds values *average+3*SD*, *average+4*SD*, *average+6*SD* and *average+7*SD* (all p>0.49).

We obtained similar results when using the algorithm of §1.6.6.2 to remove homonyms among authors instead of the fixed threshold (beta value (SEM): -0.003 (0.028), p=0.92 for the association with the increase in publications of editor’s co-authors; 0.006 (0.009), p=0.49 for the association with the increase in publications of the other authors). The thresholds values *average+3*SD*, *average+4*SD*, *average+6*SD* and *average+7*SD* did not change these results (all p>0.43).

Similar results were obtained when using the first group of co-authors of the editor identified with the algorithm of §1.6.6.1 instead of all co-authors for all threshold values with both a fixed threshold method to identify homonyms among authors (all p>0.65) or the algorithm of §1.6.6.2 (all p>0.21).

These results suggest that biases in figure 2A due to the selection of editors in the study are likely limited.

### Lack of association between the number of articles published by editor’s co-authors before the editor’s appointment and the time elapsed since the editor’s appointment

This analysis is reported in §1.10.10.

The time elapsed since the editor’s appointment at *Nature* journals was not associated with the number of articles published by editor’s co-authors before the editor’s appointment (beta value (SEM): 0.019 (0.017), p=0.26). Results were similar with the threshold values *average+3*SD*, *average+4*SD*, *average+6*SD* and *average+7*SD* (all p>0.14). Results were also similar when using the algorithm of §1.6.6.2 to remove homonyms among authors instead of the fixed threshold (beta value (SEM): 0.011 (0.015), p=0.47). The threshold values *average+3*SD*, *average+4*SD*, *average+6*SD* and *average+7*SD* did not change these results (all p>0.34) except for the threshold *average+7*SD* for which there was a trend (p=0.06). Similar results were obtained when using the first group of co-authors of the editor identified with the algorithm of §1.6.6.1 instead of all co-authors for all threshold values with either the fixed threshold method to identify homonyms among authors (all p>0.44) or the algorithm of §1.6.6.2 (all p>0.12). These results complement and extent the ones of §2.4.6. They suggest that a bias related to the selection of editors is likely limited.

There was an association between the time elapsed since the editor’s appointment at *Nature* journals and the number of articles published by the other authors before the editor’s appointment when using a fixed threshold of *average+5*SD*, *average+6*SD* and *average+7*SD* to identify homonyms among authors (p=0.05 or p=0.04). There was a trend for the threshold values *average+3*SD* and *average+4*SD* (p=0.07 and p=0.06 respectively). When using the algorithm of §1.6.6.2 to identify homonyms among authors, there was no association for the threshold values *average+3*SD*, *average+4*SD* and *average+5*SD* (all p>0.11) while there was a trend for *average+6*SD* and *average+7*SD* (p=0.09 and p=0.08 respectively). Additionally, there was no association when we used only the first group of co-authors of the editors identified with the algorithm of §1.6.6.1 both when using a fixed threshold (all p>0.18) or the algorithm of §1.6.6.2 (all p>0.27) to identify homonyms among authors. Therefore, the robustness of the association between the time elapsed since the editor’s appointment and the number of articles published by the other authors before the editor’s appointment is moderate as compared to the results of the main text. Additionally, these results are more related to the context of publication at the time of the editor’s appointment rather than to the characteristic of the editor. Thus these results do not support a bias due to the selection of editors in our study.

# Supplementary figures

## Supplementary figure 1

**N=187** job offers for publishing and editorial position at the Nature research department

**N=107** job offers for editorial positions with decisional abilities over submitted manuscripts at Nature journals included in the study

**N=77** job offers included in the analyses

**N=80** job offers for other Nature journals, other journals or positions without decisional abilities over submitted manuscripts

**N=30** job offers which are duplicates (i.e. the corresponding job offers have been posted several times on the Nature website for 17 job offers)

Supplementary figure 1: inclusion flow chart for job offers of *Nature* journals

## Supplementary figure 2


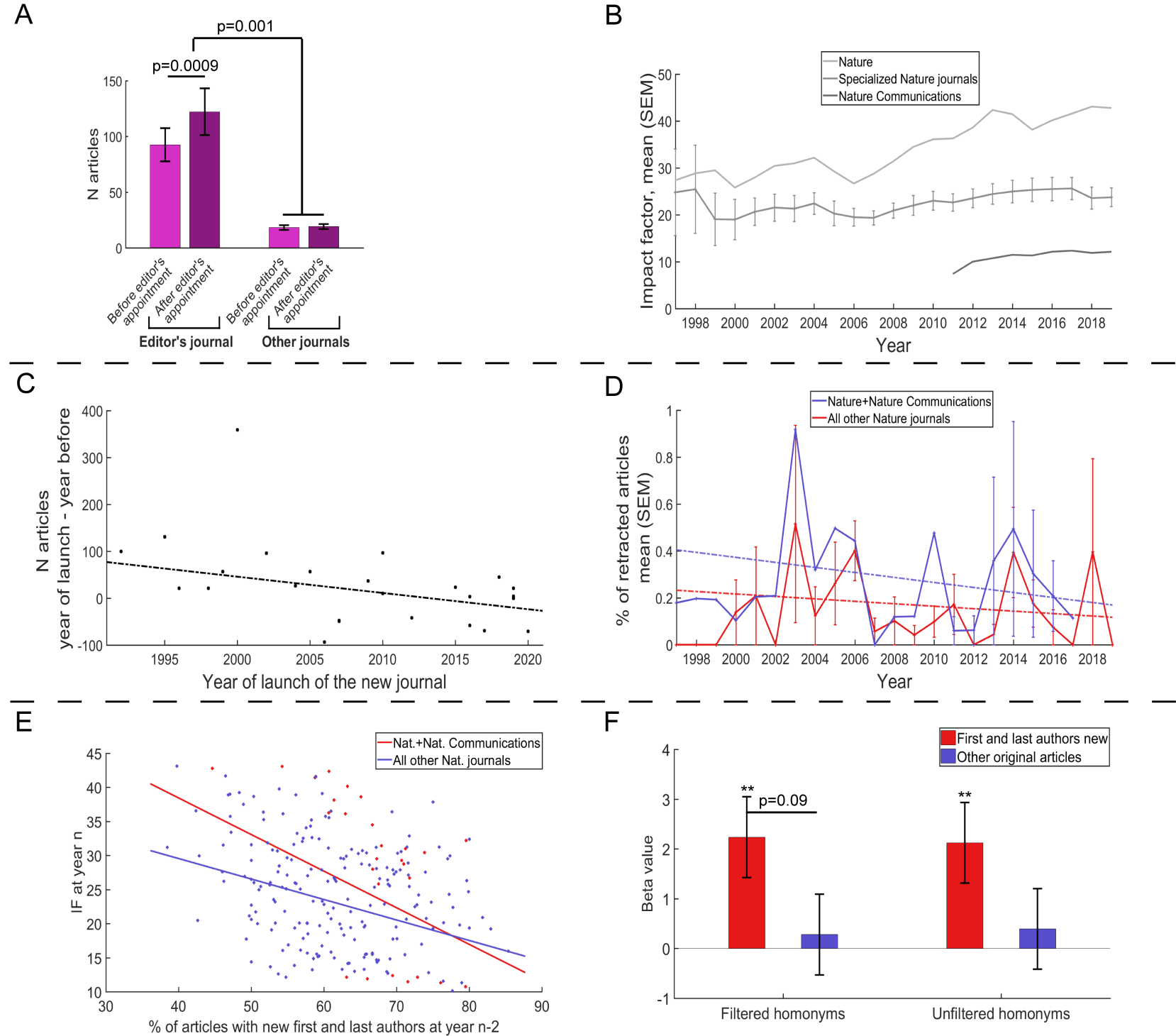


Supplementary figure 2: Total number of articles published in the editor’s journal and the other *Nature* journals (averaged across other *Nature* journals) during the two years before and the two years after the editor’s appointment at his/her current *Nature* journal by authors affiliated with the same research institutions as the editor’s before he/she joined *Nature* journals (A); Temporal evolution of the impact factor (IF) of *Nature* journals sorted by category of prestige (*Nature* is in the highest category of prestige, specialized *Nature* journals are in the intermediate category and *Nature Communications* is in the lowest category, B); Number of original articles by new authors in *Nature* and the new *Nature* journal the year of the launch of the new journal minus number of original articles by new authors in *Nature* the year before the launch (C); yearly percentage of retracted original articles in *Nature* journals (D); association between impact factor (IF) at year n and percentage of original articles published at year n-2 by new first and last authors in generalist (red) and specialized (blue) *Nature* journals (E, slope (SEM): -0.54 (0.12), p<0.00001 (red); -0.3 (0.03), p<0.00001 (blue)); Modulation by the year n of the association between N_original articles year n+1_- N_original articles year n_ and IF_year n_-IF_year n-1_ in *Nature* and *Nature Communications* for articles with new first and last authors (red) and for articles of a known first or last author (blue, F).

**: p<0.05; **: p<0.01; ***: p<0.001. These p-values are for comparisons of bars against zero (paired t-test).*

##

## Supplementary figure 3


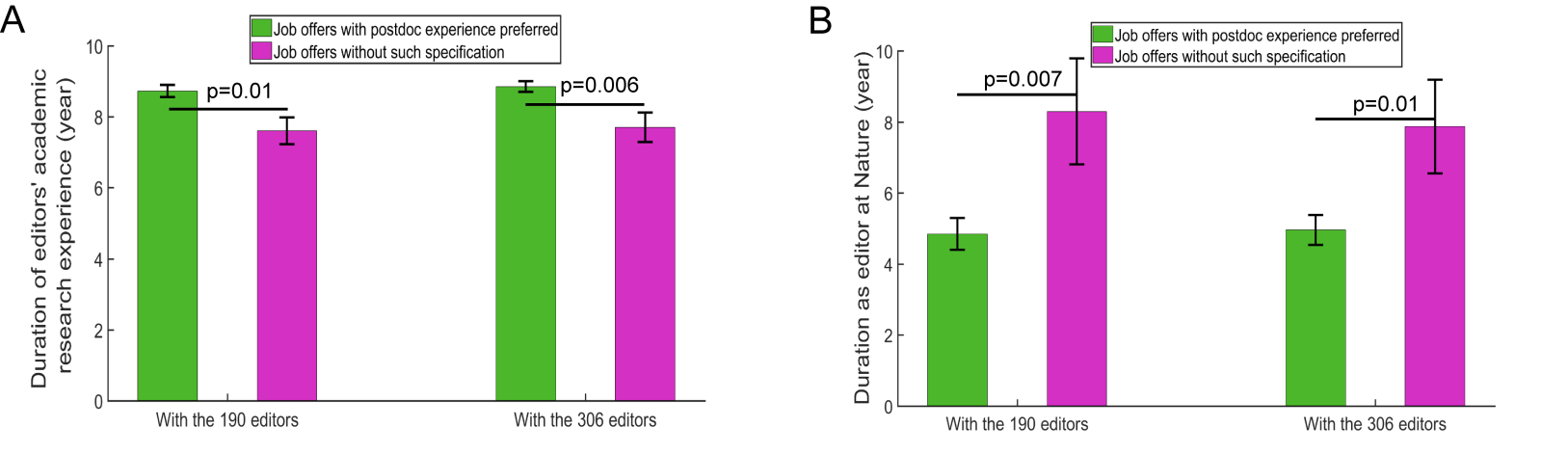


Supplementary figure 3: Average duration of academic research (A) and as editor (B) of *Nature* journals’ editorial boards issuing job offers since December 2020 for editor positions at *Nature* journals with (green) and without (violet) an explicit preference for candidates with a postdoc experience or a strong research record when considering the 190 editors included in the analyses reported in the main text (two left bars) and the 306 editors (two right bars) with an available starting date as editor at *Nature* journals compatible with the handling of submitted manuscripts before December 2020.

##

# Supplementary references

1. Gallo SA, Lemaster M, Glisson SR. Frequency and Type of Conflicts of Interest in the Peer Review of Basic Biomedical Research Funding Applications: Self-Reporting Versus Manual Detection. Sci Eng Ethics. 2016;22(1):189-97. doi: 10.1007/s11948-015-9631-7. PubMed PMID: 25649072.

2. Pacheco RL, Latorraca COC, Martimbianco ALC, Miranda E, Fontes LES, Nunan D, et al. Adherence to conflicts of interest policy in Cochrane reviews where authors are also editorial board members: a cross-sectional analysis. Res Synth Methods. 2021. doi: 10.1002/jrsm.1507. PubMed PMID: 34165922.

3. LOI n° 2012-300 du 5 mars 2012 relative aux recherches impliquant la personne humaine, 2012-300 (2012).

4. World Medical A. World Medical Association Declaration of Helsinki: ethical principles for medical research involving human subjects. JAMA. 2013;310(20):2191-4. doi: 10.1001/jama.2013.281053. PubMed PMID: 24141714.

5. Nature. Terms and conditions [04/10/2022]. Available from: <https://www.nature.com/info/terms-and-conditions>.

6. LinkedIn. LinkedIn User Agreement [04/16/2022]. Available from: <https://fr.linkedin.com/legal/user-agreement>.

7. ORCID. Terms of use [04/10/2022]. Available from: <https://info.orcid.org/terms-of-use/>.

8. RÈGLEMENT (UE) 2016/679 DU PARLEMENT EUROPÉEN ET DU CONSEIL du 27 avril 2016 relatif à la protection des personnes physiques à l'égard du traitement des données à caractère personnel et à la libre circulation de ces données, et abrogeant la directive 95/46/CE (règlement général sur la protection des données), 2016/679 (2016).

9. CHAPITRE III - Droits de la personne concernée.

10. CHAPITRE II - Principes.

11. CHAPITRE I - Dispositions générales.

12. Nature. Editorial criteria and processes 2021 & 2024 [07/13/2021 & 01/02/2024]. Available from: <https://www.nature.com/nature/for-authors/editorial-criteria-and-processes>.

13. Nature. About the Journal 2021 [07/13/2021]. Available from: <https://www.nature.com/nature/about>.

14. Bernstein P, Cohen B, MacCallum C, Parthasarathy H, Patterson M, Siegel V. PLoS biology--we're open. PLoS biology. 2003;1(1):E34. doi: 10.1371/journal.pbio.0000034. PubMed PMID: 14551925; PubMed Central PMCID: PMCPMC212705.

15. Clarivate. Journal Citation Reports: 5-Year Impact Factors 2018 [06/06/2022]. Available from: <https://support.clarivate.com/ScientificandAcademicResearch/s/article/Journal-Citation-Reports-5-Year-Impact-Factors?language=en_US>.

16. Sekara V, Deville P, Ahnert SE, Barabasi AL, Sinatra R, Lehmann S. The chaperone effect in scientific publishing. Proc Natl Acad Sci U S A. 2018;115(50):12603-7. doi: 10.1073/pnas.1800471115. PubMed PMID: 30530676; PubMed Central PMCID: PMCPMC6294962.

17. Nature. Journal metrics 2021 [07/14/2021]. Available from: <https://www.nature.com/nature-portfolio/about/journal-metrics>.

18. ORCID. ORCID. Journal of the Medical Library Association. 2017;105(2):207–8.

19. Torvik VI, Smalheiser NR. Author Name Disambiguation in MEDLINE. ACM Trans Knowl Discov Data. 2009;3(3). doi: 10.1145/1552303.1552304. PubMed PMID: 20072710; PubMed Central PMCID: PMCPMC2805000.

20. Baum T, Leßmann H, Schneider K. The Choice of Code Review Process: A Survey on the State of the Practice. Product-Focused Software Process Improvement: 18th International Conference, PROFES 2017, Proceedings. In: Science LNiC, editor.2017. p. 111–27.

21. Claxton LD. Scientific authorship. Part 2. History, recurring issues, practices, and guidelines. Mutat Res. 2005;589(1):31-45. doi: 10.1016/j.mrrev.2004.07.002. PubMed PMID: 15652225.

22. Nature. History of Nature 2021 [08/27/2021]. Available from: <https://www.nature.com/nature/about/history-of-nature>.

23. Clarivate. About Citable Items 2022 [11/08/2022]. Available from: <http://help.incites.clarivate.com/incitesLiveJCR/9607-TRS>.

24. Berenbaum MR. Impact factor impacts on early-career scientist careers. Proc Natl Acad Sci U S A. 2019;116(34):16659-62. doi: 10.1073/pnas.1911911116. PubMed PMID: 31337686; PubMed Central PMCID: PMCPMC6708304.

25. McGillivray B, De Ranieri E. Uptake and outcome of manuscripts in Nature journals by review model and author characteristics. Res Integr Peer Rev. 2018;3:5. doi: 10.1186/s41073-018-0049-z. PubMed PMID: 30140448; PubMed Central PMCID: PMCPMC6097313.

26. Siler K, Vincent-Lamarre P, Sugimoto CR, Lariviere V. Cumulative advantage and citation performance of repeat authors in scholarly journals. PLoS One. 2022;17(4):e0265831. doi: 10.1371/journal.pone.0265831. PubMed PMID: 35417471; PubMed Central PMCID: PMCPMC9007338.

27. van Lent M, Overbeke J, Out HJ. Role of editorial and peer review processes in publication bias: analysis of drug trials submitted to eight medical journals. PLoS One. 2014;9(8):e104846. doi: 10.1371/journal.pone.0104846.

28. Wilhite AW, Fong EA. Scientific publications. Coercive citation in academic publishing. Science. 2012;335(6068):542-3. doi: 10.1126/science.1212540. PubMed PMID: 22301307.

29. Teixeira da Silva JA, Dobranszki J, Bhar RH, Mehlman CT. Editors Should Declare Conflicts of Interest. J Bioeth Inq. 2019;16(2):279-98. doi: 10.1007/s11673-019-09908-2. PubMed PMID: 31016681; PubMed Central PMCID: PMCPMC6598958.

30. Nature. How Nature contributed to science's discriminatory legacy. Nature. 2022;609(7929):875-6. doi: 10.1038/d41586-022-03035-6. PubMed PMID: 36171380.

31. Nature. Nature recruitement frequently asked questions 2021 [09/17/2021]. Available from: <https://media.springernature.com/full/springer-cms/rest/v1/content/16236664/data/Springer+Nature+Recruitment+Frequently+Asked+Questions>.

32. Retraction challenges. Nature. 2014;514(7520):5. doi: 10.1038/514005a. PubMed PMID: 25279879.

33. LOI n° 2016-1691 du 9 décembre 2016 relative à la transparence, à la lutte contre la corruption et à la modernisation de la vie économique, LOI n° 2016-1691 (2016).

34. Bribery Act 2010, (2010).
