# Supplementary material for: Reputation shortcoming in academic publishing
Source: PLoS One. 2025 Apr 29;20(4):e0322012. doi: 10.1371/journal.pone.0322012 (PMC12040217; doi:10.1371/journal.pone.0322012)
Supplement: Attachment — Submitted filename: Neveu et al point by point rebuttal.pdf [file pone.0322012.s003.pdf]

Reviewer #1: The authors study the bias in editors in making decisions in the publication process. This bias is related to the authors' publications and the editor's professional circle.
Also, this manuscript shows that authors with a lesser reputation or a weaker tie to the editor have a lower chance of publication. Even this editor's odd behavior is bigger between different journals with different impact factors.

The study is very rigorous in general terms. However, the structure of the article must be modified. Some of the material proposed as supplementary should be included in the main text, and such extensive supplementary material should not be provided. On the other hand, the sections of the manuscript should be Introduction, Literary Review, and Methodology, which includes the starting hypotheses and their subsequent verification in a Discussion section. Finally, a conclusion section should be created where the most exciting aspects of the article and its weaknesses are pointed out.

**We thank the reviewer for his positive appreciation of the study. We acknowledge that the structure of the article does not fit the one of Plos One. This is due to the automatic transfer of the manuscript from Plos Biology to Plos One. The format of articles in Plos biology is different from the one of Plos One. An automatic transfer does not leave room to a reformat of the article. Accordingly, we have formatted the article to fit the structure requested by Plos One. This led us to move several elements of the supplementary materials to the main text (e.g. supplementary figure 1…). We have also reduced by 40% the supplementary materials. A substantial supplementary material is necessary to report the multiple rules addressing missing and heterogenous data in retrospective studies. Mentioning these rules allows the replicability of the study. We also had to assess the effects of these rules for the strength of the study. The table of contents of the supplementary materials allows the readers to navigate quickly and efficiently across them.**

Comments.-
1.-The authors must identify the Introduction section after the Abstract.

**We thank the reviewer for his/her point: we have added the statement "Introduction" at the beginning of the introduction section.**

2.-I'd like the authors to explain with more detail "weaker tie to the editor".

**We have reformatted the introduction and explained more in detail the statement "weaker tie to the editor" (first paragraph page 3).**

*"Thus, authors have a weaker tie to the editor when they have not worked with the editor previously, or when they have a different affiliation than the former editor's ones, or when they have a lower track record in the editor's journal family."*

3.-In the study carried out in the section "Former co-authorship as a reputational input to the editor," it is unclear to me if the authors took into account authors who had not been co-authors of the editor and were not co-authored by any editor's co-author. In this case, the editor's influence could be extended indirectly. In this section, the first time in which the authors present statistics results will be needed to indicate the type of test used.

**We thank the reviewer for highlighting this point. We did not identify what could be called "second level ties with editors": authors who were not former co-authors of the editor but who were co-authors of editor's former co-authors. The results of this investigation would be an extension of our study. We clarified this point in the manuscript (paragraph 3, page 6).**

*"We did not extend the ties to authors who never published with the editor but who were co-authors of editor's former co-authors. This extension may be viewed as an indirect, or a second level, tie with the editor."*

**We also clarified in the method section which analysis was used and which statistical test was used (paragraph 1 of the statistical analyses section, page 7).**

*"Quantitative data were analyzed with Student t-tests, Wilcoxon or the Mann-Whitney tests whenever appropriate, and qualitative data with Fisher exact or chi square tests whenever appropriate. Most analyses relied on general linear and mixed models (table 2, supp. §1.9 and §1.10). We report here the main models. The others are described in supp. §1.9 and §1.10.*

*Two types of models were designed: one for the results related to the editor's professional circle and the second one for the rate of articles by new authors or known intermediate authors."*

4.-In the section "Author's track record as a reputational input to the editor," why did the authors limit the study to new authors publishing in Nature journals? Usually, a scientist has access to the different publications of an author.

**We thank the reviewer to highlight this point. Editors of Nature journals meet every week to discuss the submitted manuscripts and editors of a given Nature journal are requested to liaise extensively with editors of other Nature journals. Thus, authors whose article(s) have been published in Nature journals have high chances to have their article been discussed in a positive manner by editors of these journals. The higher these authors have published articles in Nature journals, the higher their name has been cited in a positive manner in these weekly editorial meetings while this is less likely the case of authors who never submitted any work to Nature journals nor authors who faced editorial rejections. Therefore, there is likely a difference in reputation of authors between those who never succeeded in publishing in Nature journals as compared to those who did. We clarified this point in the method section of the manuscript (paragraph 2, page 7).**

*"The track record of authors was computed in Nature journals rather than in all journals because: homonyms are more likely when considering a higher number of journals; the editor's memory of author's names, heard in weekly editorial meetings [33] held at this type of journals or during the "extensive liaising with" editors of other Nature journals and researchers (supp. §2.2.6), is more likely to interact in the fast editorial decisions [6] than a time consuming search of the full track record of each author of an article [27]."*

5.-The period to recruit editors into the studio could be a lot longer. Have the authors looked at other time windows to recruit editors?

**We are not certain to understand the reviewer's point. Rather than the enrollment of editors in the study, we guess that the reviewer refers to the duration between the editor's appointment and the publication date of the first article handled by the editor. This was addressed in the supplementary methods. We varied that duration from 6 to 10 months in the robustness analyses. The results of the main text are reported with a duration of 8 months. We clarified that in the method section of the manuscript (paragraph 1 of the additional control analyses section, page 10).**

*"the time between the editor's appointment date and the date of publication of his/her first article handled (set to 8 months, supp. §1.6.3) which included the duration of the publication process and the duration of the editorial training provided by the journal (supp. §2.4.4),"*

**Note that a bias associated to the enrollment of the editors in the study is also addressed in the supplementary materials and referenced already in the method section of the manuscript (paragraph 1 of the additional control analyses section, page 10).**

*"the quantification of potential biases due to the selection of editors (supp. §1.10.9 and §1.10.10),"*

6.-In the box bottom in Figure 1 (in supplementary material) appears "(PhD or". I recommend that the authors translate this figure into the manuscript.

**We thank the reviewer for that suggestion. We moved that figure into the manuscript and extended it with the inclusion flow chart of journals (see figure 1). Accordingly, we reduced the supplementary materials.**

7.-In the preprocessing step of the published papers, how do the authors get the original papers?

**The full Pubmed database has been downloaded and then preprocessed. We moved the explanation of the supplementary materials to the method section of the manuscript (paragraph 3, page 6).**

*“The study was longitudinal and retrospective. It used the 31850051 published articles recorded in the PubMed database until December 12 2020 downloaded from https://ftp.ncbi.nlm.nih.gov/pubmed/baseline/ and the history of the journals’ IF available at Clarivate until 2019.”*

Reviewer #2: Thank you to the authors to submit this manuscript for review. The authors addressed the concern of publication bias based on editors' and authors' reputation. To investigate this, they reviewed three decades of publications of 33 Nature journals in physical and life sciences and they analyzed associations between publications and ties between editors and authors.

The structure of the article is very weird and accordingly very difficult to read and to understand. The first abstract is followed by a bigger abstract (?), not being clearly an introduction. This "bigger abstract" ist followed by results, than discussion and finally the method section. The authors should organize the manuscript according to the journal guidelines (https://journals.plos.org/plosone/s/submission-guidelines). Additionally, it would be recommend to use standard checklist to report research data (https://www.equator-network.org). This manuscript seems the synopsis of a PhD project (reported as a supplemental, which with 144 pages, it is of course not possible to review in this context). However, a scientific article it is not the same. The authors should focus on the problem, develope an hypothesis and test it with an appropriate sicentific method. Limitations to this should be reported in the discussion (which are missing).

**We thank the reviewer for highlighting the amount of work associated to this study. We formatted our manuscript for an initial submission to Plos Biology which has been automatically transferred to Plos One. Such a transfer is not designed to allow an in depth reformatting of a manuscript. The formatting guidelines of Plos One are different from the ones of Plos Biology.**

**The supplementary materials contain detailed methods to allow replication of our work and additional results to address multiple confounds that may alter the main conclusion of the article. Like all studies using databases of published articles, our study requires a substantial amount of preprocessing steps of the data which may create confounds in the results. These preprocessing steps are most of the time not documented even in articles published in the most renowned journals. We have substantially reduced by 40% the supplementary materials so that replication of the study may be performed with the information remaining.**

**The goal of our study was already stated at the end of the introduction (paragraph 3 of the introduction, page 2): *“To quantify a possible association between reputation and the outcome of the publication process”*. We had already included three paragraphs of limitations in the discussion and a reference to an extended view in the supplementary materials. We extended the limitations with 3 additional paragraphs (paragraphs 4 to 9 of the discussion, pages 15 and 16).**

1. Problem (and research question)
The authors reported the concern of publication bias based on editors' and authors' reputation. What do we already know (introduction)? The authors already reported apparently associations between gender of the editor and the authors (which is not described), so as faster submission process by co-authors of the editor. These concerns should be addressed in detail in the introduction. So, what is your hypothesis? If the hypothesis related to publication success, the analysis should consider rejected manuscript, which are here apparently non-available (?). If so, how planned the authors to go through without this information? Other problems should be considered: e.g. If an author has already published numerous articles across various journals, their expertise alone would inherently enhance the likelihood of a successful publication, irrespective of the editor's influence.

**We thank the reviewer for highlighting a potential confound due to rejections. This issue refers to the submission data and the review process. Submission data do not allow to control for the quality of the manuscripts. We already highlighted that point in the method section (paragraph 2 of the study design section, page 7):**

*“Of note is that publications have a more homogenous quality than submissions. Analyzing submissions requires to better control for the quality of the manuscripts which is impossible to perform over hundreds of thousands of submitted manuscripts.”.*

**Additionally, the reviewer’s request raises serious legal or methodological concerns. Submission data are not publically available. Our study requires to have access to the full name of the actors of the publication process. Transferring them to a third party raises legal concerns. An analysis made by the members of the journal raises conflicts of interests and reduces substantially the strength of the results. Nonetheless, we found two ways to address biases due to the submission behavior of authors: they were already described in the method and results sections.**

*Method section:*

*“We complemented these analyses by other linear mixed models and logistic regressions in order to control that our results were not biased by:*

- *the quantification of a potential effect of the submission behavior of authors over publications (supp. §1.11.7) to complement our results obtained with submission data,”*

*“The data of submissions of original articles we collected were the only data of submissions which were publicly available. Nonetheless, we performed analyses to quantify the impact of a change in submission behaviors of authors over publications after the editor’s appointment”*

*Results section:*

*“A change in the submission behavior of editor’s former co-authors after the editor’s appointment as compared to before can only contribute marginally to the higher increase in their publications depicted in figure 1A. Indeed, in the editor’s journal, the percentage of editor’s former co-authors who published only before the editor’s appointment was similar to the percentage of those who published only after (supp. §1.11.7 and §2.7.5). Similar results were obtained when comparing the percentage of editors whose former co-authors published only before the editor’s appointment to those whose co-authors published only after (supp. §1.11.7 and §2.7.5).”*

*“However, there was no association between IF variations and the total number of submitted articles (supp. §2.1.7).”*

**The reviewer raises also the confound of the track record of authors. Likewise the issue with submissions, this is already addressed in our analyses and reported in table 2, and in several locations both in the method and results sections:**

*Method section:*

*“In these models, we controlled for the level of the academic origins of editors by adjusting the models over track record of authors in Nature journals (table 2, supp. §1.9).”*

*Results section:*

*“Among authors with the same publishing track record in the editor’s journal before the editor’s appointment, all authors published more original articles within the two years following the editor’s appointment, compared to the two years before (Figure 1A).”*

*“To calibrate this effect, we quantified the chances of subsequent publications for authors with an established track record of publication in the editor’s journal irrespective of whether they were former co-authors of the editor.”*

**Finally, the reviewer wonders what is our hypothesis. It is included in the goal of our study stated at the end of the introduction (page 2): *“To quantify a possible association between reputation and the outcome of the publication process”*. The observational nature of our study does not allow to draw more than that. Nonetheless, the reformatting of the manuscript extended the introduction with two paragraphs (paragraphs 1 and 2, page 3) in which we precise which parameters of reputation we investigate and which question we address for these parameters.**

**Taken altogether, all the comments of the reviewer for that point are already addressed in the manuscript.**

2. Methods & results
Since the hypothesis is not precisely defined, also the method section is difficult to understand. It seems that the authors compared the number of published articles in the two years before or after a submission (with or without ties to the editor). This difference seems to be very little (ca. 1.1 vs 0.9 according to figure 1 A) and also if statistically significant, is it really the "proof" of a potential publication bias? The methods section should be "aligned" with the results sections, i.e. e.g. in each subheader of the results section should we find a subheader in the methods section, which explain the method used.

**We thank the reviewer for suggesting to add matching subheaders in the method and results sections.**

**Based on the magnitude of the bars of figure 1A, the reviewer seems skeptical ("is it really the "proof" of") about the support to a publication bias. The reviewer raises the issue of the calibration of the observed difference. First, the 31% increase in publications by editor's former co-authors after the editor's appointment means that for each set of 3 co-authors publishing one article in Nature journals, the fourth co-author publishes 2 articles in these highly competitive journals. Additionally, the magnitude of a difference cannot be interpreted without being put into the context: a difference of 0.5 second between two sprinters at the end of a 100m race does not have the same importance if the sprinters are undergraduate students running in a standard sport class or if these sprinters are running the final race of the Olympic games. This difference was already put in context in the manuscript in the second paragraph of the results section:**

*"This difference with the other authors represented 120% more chances for co-authors of the editor to publish in his/her journal after his/her appointment than the other authors (odds ratio [95% confidence interval]: 2.2 [1.5 – 3.24], p<0.0001). To calibrate this effect, we quantified the chances of subsequent publications for authors with an established track record of publication in the editor's journal irrespective of whether they were former co-authors of the editor. For each additional original article published in the two years before the editor's appointment, these authors had 161% more chances to publish another original article in this journal after the editor's appointment (odds ratio [95% confidence interval]: 2.6 [2.59 – 2.62], p<0.0001, supp. §2.1.2). The weight of the authors' relationship with an editor is thus comparable to having published one more article in the journal of the editor in author's chances to publish an original article in the editor's journal."*

**Additionally, the reviewer noticed that we analyzed Nature journals. The competition to succeed in publishing in these journals is not the same as in specialized journals with a very low impact factor. This intrinsic difference contributes to appraise the difference of figure 1A. We added a paragraph in the discussion section to highlight that point.**

*"The magnitude of some differences reported in our results and the proportion of one third of editors who had former co-authors publishing in their journal may be perceived as limited. Nonetheless, the stronger competition to publish in Nature journals than in other journals (around 8% of submissions at Nature are accepted for publication [6]) likely increases the impact of a difference even objectively small in the chances to publish in such journals."*

**Taken all together, all reviewer's concerns are already addressed in the article except adding subheadings in the method sections that match the ones of the results section.**

3. Results
The authors should report the "demographics": how many articles they analyzed? How many editors (this is there)? How many authors? The absolute number of article in difference should be given additionally to the proportions.

**All the demographics mentioned by the reviewer were already reported in our manuscript. Details of the number of editors and their characteristics in each journal were already reported in table 1 and supplementary figure 1 which is explicitly referred to in the first paragraph of the results section (page 2) and  in the inclusion criteria section of the methods (page 9) of the manuscript.**

**The number of articles analyzed were mentioned in the first paragraph of the method section (page 8) and details tailored to each statistical analysis were available in supplementary materials.**

**The number of authors and co-authors of editors were detailed in the supplementary results (paragraph 2.1.1) which were explicitly referenced in the manuscript in the first paragraph of the results section (page 2).**

**We moved elements from the supplementary materials (supplementary table 1 merged into table 1, supplementary figure 1 extended with the flow chart of journals, other figures related to articles and journals included in the analyses…) into the method and results sections, tables and figures of the main text. We also moved to the beginning of the results section (page 11) two paragraphs of results characterizing the editors included in the study. Of note is that Reviewer 1 noticed the reference to supplementary figure 1 and had a look at it.**

4. Discussion
The authors should focus first on their results. A limitation section is mandatory. Bias on the own results is not addressed. A comparison with other article focussing on the same problem is not given. Double blinding between authors and reviewers is increasing in the last years. The authos should address this in the discussion (not just name it).

**Our discussion contains already all these elements. The limitation section is three paragraphs long (paragraph 2 to 4 of the discussion, page 6). It contains potential biases to the interpretations of the results. The manuscript refers also to an extended discussion of other biases in the supplementary materials.**

**We highlighted more the results at the very beginning of the discussion (paragraph 1 of the discussion, page 14). We extended the limitations with 3 additional paragraphs (paragraphs 4 to 9 of the discussion, pages 15 and 16).**

**We also added a reference for the double blinding between authors and reviewers and put it into the context of a triple blinding with the editors (paragraph 4, page 17).**

I finally appreciate the effort of the authors to increase knowledge in the filed of the publication process. An extention of the blinding to the editors is an interesting idea. However, the authors should me more precise in the structure of the manuscript, so as to give a clear hypothesis with an appropriate design and reporting.

**We thank the reviewer for appreciating our effort.**

---

6. PLOS authors have the option to publish the peer review history of their article (what does this mean?). If published, this will include your full peer review and any attached files.

If you choose “no”, your identity will remain anonymous but your review may still be made public.

**Do you want your identity to be public for this peer review?** For information about this choice, including consent withdrawal, please see our Privacy Policy.

Reviewer #1: No

Reviewer #2: No

---

[NOTE: If reviewer comments were submitted as an attachment file, they will be attached to this email and accessible via the submission site. Please log into your account, locate the manuscript record, and check for the action link "View Attachments". If this link does not appear, there are no attachment files.]

- - - - -
For journal use only: PONEDEC3
