# Supplementary material for: Reputation shortcoming in academic publishing
Source: PLoS One. 2025 Apr 29;20(4):e0322012. doi: 10.1371/journal.pone.0322012 (PMC12040217; doi:10.1371/journal.pone.0322012)
Supplement: Attachment — Submitted filename: Neveu et al point by point answer.pdf [file pone.0322012.s004.pdf]

Journal Requirements:

When submitting your revision, we need you to address these additional requirements.

1. Please ensure that your manuscript meets PLOS ONE's style requirements, including those for file naming. The PLOS ONE style templates can be found at https://journals.plos.org/plosone/s/file?id=wjVg/PLOSOne_formatting_sample_main_body.pdf and https://journals.plos.org/plosone/s/file?id=ba62/PLOSOne_formatting_sample_title_authors_affiliations.pdf

Done

2. Note from Emily Chenette, Editor in Chief of PLOS ONE, and Iain Hrynaszkiewicz, Director of Open Research Solutions at PLOS: Did you know that depositing data in a repository is associated with up to a 25% citation advantage (https://doi.org/10.1371/journal.pone.0230416)? If you've not already done so, consider depositing your raw data in a repository to ensure your work is read, appreciated and cited by the largest possible audience. You'll also earn an Accessible Data icon on your published paper if you deposit your data in any participating repository (https://plos.org/open-science/open-data/#accessible-data).

We thank you for raising that point. Our data and programs are already stored in a public repository (last paragraph of the method section: "All programs and data that can be shared are publicly available at:

https://entrepot.recherche.data.gouv.fr/dataset.xhtml?persistentId=doi%3A10.57745%2F3QL466&version=DRAFT."). We have provided the public url of the data (https://doi.org/10.57745/3QL466) that will be activated upon full acceptance of the manuscript.

3. Please provide a complete Data Availability Statement in the submission form, ensuring you include all necessary access information or a reason for why you are unable to make your data freely accessible. If your research concerns only data provided within your submission, please write "All data are in the manuscript and/or supporting information files" as your Data Availability Statement.

Done

4. We note that you have referenced (van Lent M, Overbeke J, Out HJ. Role of editorial and peer review processes in publication bias: analysis of drug trials submitted to eight medical journals. PLoS One. 2014;9(8):e104846. doi: 10.1371/journal.pone.0104846. PubMed PMID: 25118182; PubMed Central PMCID: PMCPMC4130599.) which has currently not yet been accepted for publication. Please remove this from your References and amend this to state in the body of your manuscript: (ie "Bewick et al. [Unpublished]") as detailed online in our guide for authors http://journals.plos.org/plosone/s/submission-guidelines#loc-reference-style

We thank you for this remark. This reference is published on the Plos One website and has 62 citations: https://journals.plos.org/plosone/article?id=10.1371/journal.pone.0104846. Are you certain that this reference is not accepted for publication? On our side, we have edited the reference to remove the statement "PubMed PMID: 25118182; PubMed Central PMCID: PMCPMC4130599."

5. Please include captions for your Supporting Information files at the end of your manuscript, and update any in-text citations to match accordingly. Please see our Supporting Information guidelines for more information: http://journals.plos.org/plosone/s/supporting-information.

We have added a statement after the Acknowledgment section:

"Supporting information

The file "S1 Supplementary materials and figures" contains complementary information about the method, additional results, tables and figures. References with § in the main text refer to the paragraph in the content table of S1."

Please review your reference list to ensure that it is complete and correct. If you have cited papers that have been retracted, please include the rationale for doing so in the manuscript text, or remove these references and replace them with relevant current references. Any changes to the reference list should be mentioned in the rebuttal letter that accompanies your revised manuscript. If you need to cite a retracted article, indicate the article's retracted status in the References list and also include a citation and full reference for the retraction notice.

Additional Editor Comments (if provided):

Journal office requests:

*** Please update the title to meet PLOS ONE submission guidelines**

We have added the short title as requested in the submission guidelines, checked the length of the title and short titles and followed the formatting guidelines of the example of the title page.

*** On Page 16 there is extended discussion of "blinding", but this should be revised to "masking" or "anonymised", as appropriate**

We replaced blinding by masking or anonymized whenever appropriate.

"However such regulations do not address a role of an author's track record or the journal's impact factor in the publication process. As in clinical trials in which blindness of all actors is the gold standard to control for biases in any type of assessment involving human beings, anonymizing the manuscript may mitigate the role of the author's track record, the journal's IF and the editors' or authors' professional circle. Masking of authors' names to reviewers is a submission option in journals. Extending the masking of authors' names and affiliations to editors and making it systematic may better address reputation biases. Such a masking, so called triple blinding, is already implemented in a limited number of journals in philosophy and quality improvement. It is not straightforward and requires substantial adaptations from authors and journals. Authors would need to remove elements of their manuscript and cover letter that would allow to identify its origin. This requires from authors to mention neutrally their previous work. Elements that could not be removed (e.g. names and funding information) would be placed in a specific section of the manuscript that could be automatically masked by the submission system. It would be disclosed only after the final editorial decision. Editorial assistants would assess the adequate anonymization of the manuscript before editors would have access to it. Artificial intelligence implemented in the submission system might help in this process."

*** The authors indicate that the journals needed to belong to the "Nature Springer" group, but it should be designated "Springer Nature"**

We thank you for highlighting that error. We corrected it in the main text (page 5) and in the supplementary materials

*** The authors' search is limited to Pubmed, and that presents an issue when looking at the publication record of editors working on journals like 'Nature Physics'. This should be discussed as a limitation (it is currently only touched on briefly in the Results section)**

We thank you for highlighting this issue. We moved to the main text the paragraph dedicated to that issue which was in the supplementary materials.

“Because research topics in physical sciences are only partially referenced in the PubMed database, the publication track record of editors of Nature journals in those fields may be underestimated. To circumvent that, we also collected the time spent in academia by editors. Results observed with the publication track record were in agreement with those observed with the editor’s time spent in academia.”.

Reviewers’ comments:

Reviewer #1: The authors have improved the article and have satisfactorily answered my questions.
The article rigorously analyses the bias in different journals regarding the peer review, analyzing aspects collateral to the context of the submitted manuscript's content.

We thank reviewer 1 for his/her contribution in improving the manuscript.

Reviewer #2: Thank you to the authors for this review and the thorough response to the previous comments. The authors thoroughly explored the research question and developed a clear understanding of the concepts presented. However, some of these concepts might be difficult for a reader encountering the manuscript for the first time. The revision has made some of these concepts easier to understand. All the questions were addressed. The flow of the whole manuscript could be still improved, sometimes being written to complicated and mixed in some concepts. For example, in the introduction section, to postulate the hypothesis the authors stated: "We quantified a possible association at the editorial level between reputation and the outcome of the publication process. We focused on ties between authors, editors and the journal’s impact factor (IF)." which, to me, sounds somewhat "cryptic". I would have preferred a clearer and more straightforward statement, such as: "We hypothesized an association between the number of publications among authors and editors with ties compared to those without ties". After that, the authors should present the objective of the study. However, it seems that what follows is a mix of the aim and the methods used to achieve it. Finally, I believe the manuscript is understandable, and I acknowledge that the writing style might be a matter of personal preference.

We thank reviewer 2 for his/her suggestion. We have modified the second part of the introduction accordingly.

“That reputation may interact with the outcome of the publication process at the editorial level is supported by several psychological mechanisms: the use of heuristics, such as reputation, in fast decisions, a characteristic featured in editorial decisions; the poor sensibility of editors to problems in the publication process; the sensitivity of journals to reputation; the subjective nature of editorial criteria for publication.

We hypothesized that authors’ ties with editors are associated with the number of their publications. We assessed whether this is related to three aspects of authors’ reputation in the eyes of the editor: a past collaboration with the editor, an affiliation to one of the editor’s former research affiliations, and to which extent the editor may have heard about an author during the editorial meetings where most of the submitted articles are discussed. We collected three variables to capture these aspects: the number of publications of editor’s former co-authors in the editor’s journal, the number of articles published in the editor’s journal whose authors were affiliated with one of the editor’s former research

affiliations, the track record of authors in the editor's journal family. Thus, authors have a weaker tie to the editor when they have not worked with the editor previously, or when they have a different affiliation than the former editor's ones, or when they have a lower track record in the editor's journal family."
