# Supplementary material for: Reputation shortcoming in academic publishing
Source: PLoS One. 2025 Apr 29;20(4):e0322012. doi: 10.1371/journal.pone.0322012 (PMC12040217; doi:10.1371/journal.pone.0322012)
Supplement: Attachment — Submitted filename: Neveu et al point by point response after revisions.pdf [file pone.0322012.s005.pdf]

Reviewer #3: I have enjoyed reading this interesting work. The authors address a problem that currently exists in relation to professional editors with little academic experience. The results clearly show that their selection as editors is related to the increase in publications by the co-authors of these same editors. An increase much higher than that observed under other situations such as gender. All this points to a clear lack of experience of the professional editors who have been analyzed, which has repercussions on scientific publication in the journals analyzed. Their lesser academic experience is possibly taken advantage of by their co-authors and the same publishers to increase the number of publications by specific authors, the co-authors of these professional editors and the authors of articles that have already been published in the journals analyzed. As a future line of research, the authors could verify the relationship that exists between these same results and those that would be obtained in journals with academic editors who have greater academic and/or professional prestige.

We thank the reviewer for his enthusiasm for our study and his suggestion of future research. Studying academic editors introduces additional confounds related to the concomitant activity of research and editing: one activity may influence the other and vice versa. A specific method should be designed to control for this effect and would be the topic of another study.

Reviewer #4: The article examines the factors influencing the editor's decision to accept or reject submitted manuscripts. These factors can impact the quantity of scientific output for both the author and the editor. This is an interesting topic that is of significant interest to the scientific community. Nevertheless, the minor revisions mentioned below may help improve the article.

We thank the reviewer for his overall appreciation of the study.

Introduction:
- The introduction is well-structured and provides an overview of the publication process and the role of reputation. With some minor revisions, it will be even stronger. At the end of the introduction, the coherence of the content diminishes, resulting in the reader anticipating a summary of the research objectives, innovations, and contributions.

We thank the reviewer for suggesting this improvement. We moved the last paragraph of the introduction and a part of the previous paragraph to the description of the statistical analyses in the method section. In the meantime, we have added a summary at the end of the introduction:

"The role of reputation in the publication process has attracted little attention. We hypothesized that authors' ties with editors are associated with the number of their publications. Analyzing three decades of publications of 33 Nature journals in physical and life sciences, we show that authors with less reputation or a weaker tie to the editor have substantially lower chances to publish in the editor's journal. This encompasses three aspects of authors' reputation in the eyes of the editor: a past collaboration with the editor, an affiliation to one of the editor's former research affiliations, and the author's publication track record in Nature journals. These differences in the publication outcome are associated with the scientific experience of the editor and amplified by variations in the impact factor (IF) of the journal. Some of these differences changed after the establishment of a non-financial COI policy by journals. We controlled for major confounding factors such as the submission behavior of authors, the academic origin of editors or the launch of a new journal. We conclude by suggesting further changes in order to mitigate the importance of reputation in the publication process. "

And the paragraph moved to the description of the statistics in the method section leads to:

"We focused on three variables which captured ties between authors and editors: the number of publications of editor's former co-authors in the editor's journal, the number of articles published in the editor's journal whose authors were affiliated with one of the editor's former research affiliations, the track record of authors in the editor's journal family. Thus, authors have a weaker tie to the editor when they have not worked with the editor previously, or when they have a different affiliation than the former editor's ones, or when they have a lower track record in the editor's journal family.

These three variables may uncover several confounds unrelated to reputation or to editors: the submission behavior of authors, the academic origins of editors, the content of articles are some of them. We tackled these issues by controlling them with the study design, by estimating the biases of these confounds over our results and by strengthening in three ways the tie between the editor and the three variables. First, for publications of editor's former co-authors and authors affiliated to one of the editor's former affiliations, we investigated differences between the periods before and after the editor's appointment at his/her journal. We also quantified the interaction with the setup, by the editor's journal, of a non-financial COI policy addressing ties between authors and editors. Second, because the impact factor (IF) of journals may influence editors, we investigated whether the variations in the IF of the editor's journal were associated with the publications of authors with different track records in the family of journals of the editor. Third, we tested whether the observed results were associated with the scientific or academic experience of the editor."

Methods:
- This section is organized in a manner that narrates the progression of the research. Concerning supplementary methods all procedures are presented in a logical sequence that enables the reader to trace the gradual development and nuances of the study.

We thank the reviewer for his appreciation of our presentation of the methods.

Data analyses: Preprocessing step:

- It is stated, "Note that an author may share the affiliation of the editor without being one of his/her former co-authors." This statement is not understandable to me, at least. Why might an author mention the editor's institutional affiliation without having a contribution to the authorship?

We thank the reviewer for raising that point. We clarified this statement with an example:

"Note that an author may share one of the former research affiliations of the editor without being one of his/her former co-authors: for instance, in a given university, a researcher publishing an article in neuroscience in the editor's journal may have never collaborated with the editor even though this latter has also worked at the same university and also published articles in neuroscience."

- In "Former editor's research affiliation as a reputational input to the editor" the comparison was conducted using the main type of t-test (Independent or Paired Samples t-test), which is important to mention.

We precised the type of t-test we used:

"These two numbers were compared with a paired sample Student t-test."

- In " Interaction with the setup by journals of a non-financial conflicts of interest policy" it is stated "We complemented the linear mixed model involved in the analysis of the difference . . ." It is recommended to provide a rationale for the use of the linear mixed model.

We detailed the rationale of using a linear mixed model:

"The use of a linear mixed model allows to account for the unbalanced nature of the data: the number of editor's former co-authors differs between editors and the number of publications differs between the former co-authors of a given editor. The same considerations apply for the other authors over the periods of interest around the editor's appointment date."

Discussion:

- It is advisable to consider the ethical implications of the research findings in the conclusion.

We thank the reviewer for this suggestion. We added a statement in the conclusion section:

"That journals clarify on their website the situations they consider as non-financial conflicts of interests and homogenize this list between journals would contribute to strengthen the credibility of the featured fairness of the publication process."

Final Remarks:

Reputation in the ecosystem of scientific communication is a phenomenon influenced by various factors. Editors' professional circle and authors' track record factors influence the process of scientific production. However, this claim cannot be supported by estimation alone and requires further research, which this article has successfully undertaken. It must be acknowledged that managerial positions in higher education institutions can also influence scientific publications. The findings of this research are beneficial for audiences interested in the field of scientific publishing.

We thank the reviewer for raising a potential confounding factor related to the position of authors. We added a statement in the discussion:

"The launch of a new journal, the reputation of reviewers, a potential pressure of editor's former co-authors after the editor's appointment, the author's position in the academic research institution are some of them. Author's position in the academic research institution is partially captured by the authorship rank in the article: authors with an established position are more likely to be last author than authors with a postdoc position who are likely to be first author. Nonetheless results reported in Fig. 2C, additional analyses and previous results suggest that several confounding factors such as the launch of a new journal, the scientific content of manuscripts, the author's position in an academic research institution or the recruitment of new editors do not contribute to decrease the differences in publication records between authors with different ties to the editor (S1 §2.2.3 to §2.2.5)."

Reviewer #5: The paper 'Reputation shortcoming in academic publishing' used an example of Nature family journals to detect possible biases in accepting decision made by editors toward manuscripts submitted by certain groups of authors. Specifically, the paper studied former co-authors of editors, authors from former editors' affiliations, authors with papers in Nature journals, the influence of impact-factors variations of journals. Given that the topic attracts high attention from the perspective of transparency in academic publishing, on the one hand, and poor knowledge on relationship between authors, editors and reviewers, on the other hand, the paper can make significant contribution in our understanding of hidden publishing processes.
As it is seen, the paper has already been reviewed and seems to be balanced, well-structured and detailed. Thus, I do not have many comments. Furthermore, I admire the authors' comprehensive and thoroughly prepared appendix that can be considered as a good model for other researchers in the processes of describing methodology.

We thank the reviewer for appreciating our effort to provide all elements necessary to replicate the study.

My general comment concerns some difficulties in reading, as many details become clear only

with further reading or addressing to the appendix. Thus, perhaps, it will be useful to:
1) explain the notion of "track record" on first mention;

We thank the reviewer for that suggestion. We added a definition at the end of page 3:

"…with authors, editors or reviewers. An author's track record was defined as the number of publications of this author within a set of journals and published over a specific period."

2) explain all abbreviations (e.g., IF, SD…)

We thank the reviewer for highlighting our missing. IF, SD and SEM have been explained. All other abbreviations relate to figures and supplementary materials and are imposed by Plos One.

"This difference is associated with the scientific experience of the editor and amplified by variations in the impact factor (IF) of the journal."

"…only one editor satisfied this criterion, yielding a standard deviation (SD) equal to 0"

"…(C, slope (standard error of the mean (SEM)): -1.34 (0.29)…"

3) describe in more detail the recent concept of non-financial conflict of interests. As it was a landmark in achieving higher degree of transparency, I believe the readers will be interested in specific points of its application in academic journals.

We thank the reviewer for this suggestion. We added the description of the principle defining a non-financial conflict of interest and how it applies in our current study in the introduction:

"Non-financial conflicts of interests are interests that do not engage financial transactions but could "undermine the objectivity, integrity and the value of a publication through a potential influence on the judgments and actions of authors". Scientific journals have set policies to address this issue: some journals state explicitly as such conflicts relationships between authors and the other actors of the publication process while other journals do not when it comes to the question of their own editors."

One of the conclusions of the paper concerns promotion of triple blind model in peer-review. However, the opposite 'open identities' model is currently also being promoted. Thus, it will be interesting to know the authors' attitude to this model as well. Can it be helpful to eliminate the detected biases in decision-making by editors? By the way, the authors selected the journal with single-blind model for their submission.

We thank the reviewer for raising the question of open peer review models. We made it more explicit in the discussion section and explained how the triple blinding and open peer review models may be complementary to benefit from the advantages of the two:

"The name of the handling editor could be systematically disclosed in the article to improve transparency. This disclosure may be set within the framework of an open peer review model of publication in which reviewers, editors and authors' names are disclosed to one another."

"The triple blinding and open peer review models may be complementary: the blinding may be set until the final editorial decision and the names and affiliations of the handling editor, the reviewers and authors would be disclosed to one another after the final editorial decision whatever this latter is. One may expect that editors, authors and reviewers may thus be more careful about conflicts of interests in their assessment of the work of the other actors of the publication process. "

The limit of open review models in addressing some of the biases we show was already mentioned in the paragraph where triple blinding is mentioned:

“However such regulations do not address a role of an author’s track record or the journal’s impact factor in the publication process.”

We acknowledge that Plos One has a single-blind model of peer review. We believe that biases in the scientific enterprise are a critical issue which is a matter of concern for everybody in the society and not only in the scientific community. We thus selected Plos One for publication because of its generalist nature which allows a potential to capture attention of a wider readership than a specialized journal. That articles can be published as open access in Plos One is another advantage.
